# MRI biomarker assessment of neuromuscular disease progression: a prospective observational cohort study

**DOI:** 10.1016/S1474-4422(15)00242-2

**Published:** 2016-01

**Authors:** Jasper M Morrow, Christopher D J Sinclair, Arne Fischmann, Pedro M Machado, Mary M Reilly, Tarek A Yousry, John S Thornton, Michael G Hanna

**Affiliations:** a MRC Centre for Neuromuscular Diseases, UCL Institute of Neurology, London, UK; b Neuroradiological Academic Unit, UCL Institute of Neurology, London, UK; c Lysholm Department of Neuroradiology, National Hospital for Neurology and Neurosurgery, London, UK; d Department of Radiology, University of Basel Hospital, Basel, Switzerland

## Abstract

**Background:**

A substantial impediment to progress in trials of new therapies in neuromuscular disorders is the absence of responsive outcome measures that correlate with patient functional deficits and are sensitive to early disease processes. Irrespective of the primary molecular defect, neuromuscular disorder pathological processes include disturbance of intramuscular water distribution followed by intramuscular fat accumulation, both quantifiable by MRI. In pathologically distinct neuromuscular disorders, we aimed to determine the comparative responsiveness of MRI outcome measures over 1 year, the validity of MRI outcome measures by cross-sectional correlation against functionally relevant clinical measures, and the sensitivity of specific MRI indices to early muscle water changes before intramuscular fat accumulation beyond the healthy control range.

**Methods:**

We did a prospective observational cohort study of patients with either Charcot-Marie-Tooth disease 1A or inclusion body myositis who were attending the inherited neuropathy or muscle clinics at the Medical Research Council (MRC) Centre for Neuromuscular Diseases, National Hospital for Neurology and Neurosurgery, London, UK. Genetic confirmation of the chromosome 17p11·2 duplication was required for Charcot-Marie-Tooth disease 1A, and classification as pathologically or clinically definite by MRC criteria was required for inclusion body myositis. Exclusion criteria were concomitant diseases and safety-related MRI contraindications. Healthy age-matched and sex-matched controls were also recruited. Assessments were done at baseline and 1 year. The MRI outcomes—fat fraction, transverse relaxation time (T2), and magnetisation transfer ratio (MTR)—were analysed during the 12-month follow-up, by measuring correlation with functionally relevant clinical measures, and for T2 and MTR, sensitivity in muscles with fat fraction less than the 95th percentile of the control group.

**Findings:**

Between Jan 19, 2010, and July 7, 2011, we recruited 20 patients with Charcot-Marie-Tooth disease 1A, 20 patients with inclusion body myositis, and 29 healthy controls (allocated to one or both of the 20-participant matched-control subgroups). Whole muscle fat fraction increased significantly during the 12-month follow-up at calf level (mean absolute change 1·2%, 95% CI 0·5–1·9, p=0·002) but not thigh level (0·2%, −0·2 to 0·6, p=0·38) in patients with Charcot-Marie-Tooth disease 1A, and at calf level (2·6%, 1·3–4·0, p=0·002) and thigh level (3·3%, 1·8–4·9, p=0·0007) in patients with inclusion body myositis. Fat fraction correlated with the lower limb components of the inclusion body myositis functional rating score (ρ=–0·64, p=0·002) and the Charcot-Marie-Tooth examination score (ρ=0·63, p=0·003). Longitudinal T2 and MTR changed consistently with fat fraction but more variably. In muscles with a fat fraction lower than the control group 95th percentile, T2 was increased in patients compared with controls (regression coefficients: inclusion body myositis thigh 4·0 ms [SE 0·5], calf 3·5 ms [0·6]; Charcot-Marie-Tooth 1A thigh 1·0 ms [0·3], calf 2·0 ms [0·3]) and MTR reduced compared with controls (inclusion body myositis thigh −1·5 percentage units [pu; 0·2], calf −1·1 pu [0·2]; Charcot-Marie-Tooth 1A thigh −0·3 pu [0·1], calf −0·7 pu [0·1]).

**Interpretation:**

MRI outcome measures can monitor intramuscular fat accumulation with high responsiveness, show validity by correlation with conventional functional measures, and detect muscle water changes preceding marked intramuscular fat accumulation. Confirmation of our results in further cohorts with these and other muscle-wasting disorders would suggest that MRI biomarkers might prove valuable in experimental trials.

**Funding:**

Medical Research Council UK.

## Introduction

In experimental trials, objective, responsive, and valid outcome measures are needed to test treatment efficacy. Neuromuscular disorders are common,^1^, ^2^ disabling, muscle wasting disorders, largely without proven therapy. The increasing identification of tractable targets that suggest new therapies drives a concomitant need for effective trial outcome measures.^3^ Responsive outcome measurement in neuromuscular disorders is challenging: longitudinal progression is typically slow, so disease progression might be masked by age-related changes^4^ or measurement variation, and new therapies are more likely to halt or slow progression than reverse established tissue damage. This limits outcome measure responsiveness, expressed as the standardised response mean (SRM);^5^ SRM is a key determinant of study power, with an inverse square relation to required sample size for a stated statistical power by Lehr's formula (panel 1).^6^ Outcome measure validity in terms of correlation against measures held to be valid markers of patient function or experience, such as relevant neuromuscular disorder functional rating scales, must also be established.

The shortcomings of established outcome measures were exemplified in the UK–Italian ascorbic acid trial^6^ in patients with Charcot-Marie-Tooth disease 1A. The trial showed no treatment benefit, and the placebo group natural history data showed only low primary outcome measure responsiveness (Charcot-Marie-Tooth disease Neuropathy Score SRM=0·19). The study therefore might have been underpowered, with the negative result suggesting type II error. Increasing group sizes to compensate for low responsiveness is challenging in rare diseases, and a pressing need exists to identify and validate adequately responsive outcome measures for neuromuscular disorder clinical trials.^7^

Acute or early pathological muscle changes, whether due to denervation, dystrophy, or inflammation, typically involve tissue water changes, whereas chronic disease is characterised by intramuscular fat accumulation. Intramuscular fat accumulation is the final common pathological pathway for many different primary genetic and acquired neuromuscular disorders. MRI Dixon fat-water imaging,^8^ which quantifies tissue fat content on a 0–100% fat-fraction scale, has been applied to skeletal muscle in neuromuscular disorders.^9^, ^10^, ^11^, ^12^, ^13^ Muscle MRI transverse magnetisation relaxation time (T2) and magnetisation transfer ratio (MTR) are sensitive to changes in muscle water distribution^14^, ^15^ and lipid content. Potential demonstrated in these studies to quantify both early or acute and chronic pathology non-invasively and objectively suggests these markers might be ideal candidate outcome measures for neuromuscular disorders. We previously showed their reliability and normative age dependencies and sex dependencies in healthy volunteers.^16^ However, before application in trials, their validity and responsiveness as disease markers in specific neuromuscular disorders must be established.

We aimed to test the hypothesis that these indices are valid and responsive markers of disease progression using clinical, myometric, and MRI assessments at baseline and 12 months. We studied two deeply phenotyped cohorts with Charcot-Marie-Tooth disease 1A and inclusion body myositis—representing neurogenic and myopathic, and genetic and acquired, conditions with variable disease progression rates—and age-matched and sex-matched controls. We aimed to examine the validity of MRI-quantified intramuscular fat accumulation as an outcome measure by direct correlation with clinical and myometric measures and establish the sensitivity of MRI measures to early muscle water changes before substantial intramuscular fat accumulation.

## Methods

### Study design and participants

**Figure 1:**
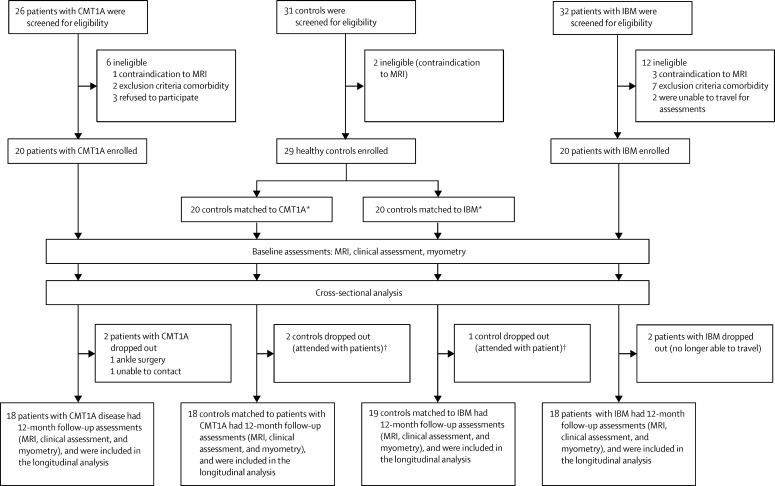
Flow chart of patient assessments and dropout CMT1A=Charcot-Marie-Tooth 1A. IBM=inclusion body myositis. *11 controls were common to both disease control groups. †Three controls dropped out because they had undertaken baseline assessments accompanying patients who dropped out before repeat assessments.

We did a prospective longitudinal observational study of patients attending the inherited neuropathy or muscle clinics at the Medical Research Council (MRC) Centre for Neuromuscular Diseases at the National Hospital for Neurology and Neurosurgery, London, UK (figure 1). Inclusion criteria were genetic confirmation of the chromosome 17p11·2 duplication for patients with Charcot-Marie-Tooth disease 1A, and classification as pathologically or clinically definite by MRC criteria^17^ for patients with inclusion body myositis, and being aged 17 years or older (as the study was based in an adult hospital). Exclusion criteria were concomitant diseases and safety-related MRI contraindications (appendix). Patients meeting these inclusion criteria and attending the clinics were invited to participate. Healthy participants were enrolled as controls in two overlapping subgroups comprising 20 participants each, one matched for age, sex, weight, and body-mass index distribution to the Charcot-Marie-Tooth disease 1A group and one matched for the same variables to the inclusion body myositis group. Controls were recruited in the first instance from spouses, friends, and relatives of the patients, who underwent assessments on the same day as the respective patient (relatives of patients with Charcot-Marie-Tooth disease 1A were included only if genetic testing was negative). Because patients with inclusion body myositis group are older men (usually older than 45 years),^18^ the controls recruited were not well matched to those patients, and the youngest six individuals in the inclusion body myositis control subgroup were replaced by six healthy individuals recruited from hospital and research staff whose ages fell within the specific demographics of the inclusion body myositis group. Clinical assessments and myometry were done at the MRC Centre for Neuromuscular Diseases, UCL Institute of Neurology, UK. MRI was done at the National Hospital for Neurology and Neurosurgery, London, UK. Cross-sectional MRI data from all of the control participants, combined with data from other participants scanned using the same MRI protocol, have been reported previously.^16^

The study was approved by the local ethics committee and all participants provided written informed consent at enrolment.

### Procedures

The following assessments were done in all patients: medical history, neurological examination, bedside strength examination using MRC grading (appendix), and SF-36 quality of life questionnaire.^19^ Patients with Charcot-Marie-Tooth disease 1A were assessed with the Charcot-Marie-Tooth examination score (version 2; CMTES),^20^ a 28-point score based on signs and symptoms where 28 is very severely affected. Patients with inclusion body myositis were assessed with the inclusion body myositis functional rating scale (IBMFRS), a 40-point score where 0 is most functionally impaired.^21^ Patients and controls underwent detailed lower limb myometry on a HUMAC NORM dynamometer (CSMi, MA, USA; see appendix for full assessment protocol).

Participants were examined lying feet-first and supine in a 3T MRI scanner (TIM Trio, Siemens, Erlangen, Germany). The quantitative MRC Centre MRI protocol was developed and included fat fraction, transverse relaxation time (T2), and magnetisation transfer ratio (MTR) measurement. These parameters were analysed during the 12-month follow-up, by measuring correlation with functionally relevant clinical measures, and for T2 and MTR, sensitivity in muscles with fat fraction less than the 95th percentile of the control group. The following measurements were done: 3-point-Dixon fat-fraction quantification resulting in fat-fraction maps expressed as percentage fat (0–100%),^8^ non-fat-suppressed T2 measurement by dual-contrast turbo-spin-echo imaging, and MTR imaging requiring two 3D-FLASH images with and without a magnetisation transfer pre-pulse with radiofrequency field (B1) non-uniformity correction resulting in MTR maps with values expressed by convention in percentage units (0–100 pu).^22^ Sequence details and variables are listed in the appendix; the total acquisition time per participant per visit was about 35 min.

### Statistical analysis

The number of patients recruited from each of the disease groups was based pragmatically on the number of patients known to have the disease of interest and eligible for enrolment. 20 patients in each disease group were deemed sufficient to identify trends in MRI variables and provide data useful to inform statistical power estimates in future studies.

**Figure 2:**
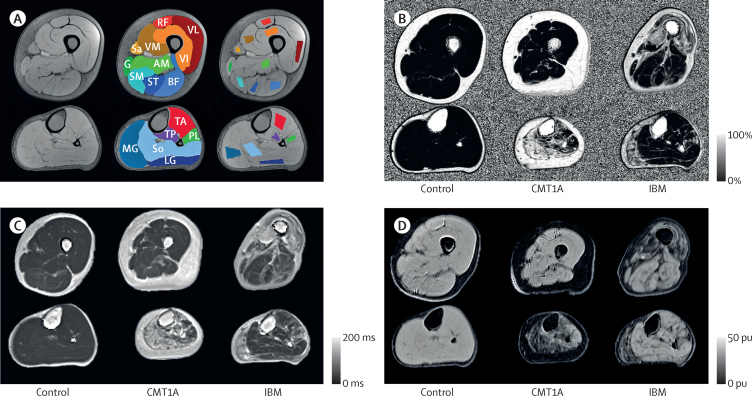
Regions of interest and sample axial images of left lower limb In each panel the upper row shows the mid-thigh level and the lower row shows the mid-calf level. (A) Unprocessed Dixon sequence (echo time=3·45 ms) in a healthy control (left), with overlaid whole muscle regions of interest (centre) and small regions of interest (right) for the same participant. (B) Fat-fraction map of a participant from each group. (C) Transverse relaxation time (T2) map of a participant from each group. (D) Magnetisation transfer ratio map of a participant from each group. RF=rectus femoris. VL=vastus lateralis. VM=vastus medialis. VI=vastus intermedius. Sa=sartorius. G=gracilis. AM=adductor magnus. SM=semimembranosus. ST=semitendinosus. BF=biceps femoris. TA=tibialis anterior group. MG=medial head of gastrocnemius. So=soleus. TP=tibialis posterior. PL=peroneus longus. LG=lateral head of gastrocnemius. pu=percentage units.

A single observer (a radiologist with 4 years of specialist experience in neuromuscular imaging) masked to all clinical details including diagnosis defined whole muscle and small regions of interest (ROIs) for each participant on single slices (appendix) for all muscles at mid-thigh and mid-calf levels from an unprocessed Dixon acquisition (echo time=3·45 ms) using ITK-SNAP software (figure 2).^23^

The small ROIs were transferred to the co-registered fat fraction, T2, and MTR variable maps. For each muscle, mean T2, mean fat fraction, and mean MTR were recorded from the small ROIs, and mean fat fraction and muscle cross-sectional area (CSA) were recorded from the whole muscle ROIs. A systematic bias was noted in T2 measurements after a routine scanner software upgrade necessitating a minor correction to post-upgrade values (appendix). For each participant, individual muscle values were combined into a summary measure calculated for each variable for all muscles (left and right limb) at thigh level and at calf level, and for relevant functional muscle groups (quadriceps, hamstrings, anterior tibial compartment, and triceps surae) for left and right limbs separately. Summary measures were calculated for small ROIs as the mean of the individual muscle values, and for whole muscle ROIs as the mean of individual muscle values weighted by cross-sectional area. To assess early pathological changes in patients' muscles for which intramuscular fat accumulation lay within the normal healthy range, additional analyses included only muscles with mean fat fraction less than the 95th percentile of the healthy control data for thigh and calf muscles separately. As a measure of the functional muscle CSA, the metric remaining muscle area was calculated with the following equation, where CSA and fat fraction refer to the whole muscle ROI values:

$$\text{Remaining}\;\text{muscle}\;\text{area}=\frac{\text{CSA}\times (100-\text{fat}\;\text{fraction})}{100\%}$$

Longitudinal changes were quantified on a muscle-by-muscle, variable-by-variable basis, and combined in the same way as for cross-sectional data to create separate all-muscle summary variables at thigh level and calf level.

Statistical analyses were done with IBM-SPSS Statistics (version 20). At baseline, cross-sectional differences between each patient group and their respective matched controls were assessed for each measure using two-tailed *t* tests. Correlations between measures at baseline were assessed with Pearson or Spearman correlation as appropriate. Missing data were excluded from the analysis. Differences between baseline and follow-up values for each measure for each patient group were assessed with paired *t* tests, and two-tailed *t* tests compared absolute change over 12 months between the patient and respective matched-control groups. Multivariate linear regressions were done to assess the dependence of T2 and MTR upon disease group membership in muscles without substantial intramuscular fat accumulation (defined as fat fraction less than the control group 95th percentile), while adjusting for the influence of residual fat fraction as a covariate.

### Role of the funding source

The study funders had no role in the study design, data collection, data analysis, data interpretation, or writing of the report. The corresponding author and all coauthors had full access to all the data in the study and had final responsibility for the decision to submit for publication.

## Results

**Figure 3:**
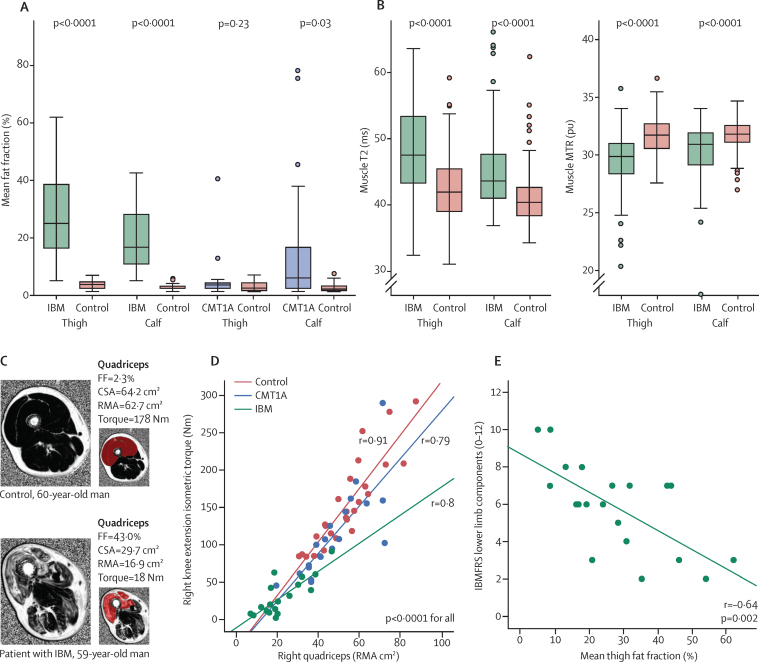
Cross-sectional data (A) Overall fat fraction is significantly increased at both thigh and calf level in patients with inclusion body myositis and at calf level in patients with CMT1A compared with matched controls. Boxes represent median and IQR, whiskers show range, and filled circles are outliers. (B) Combination of all muscles without substantial intramuscular fat accumulation shows that muscle T2 is increased and MTR is reduced in muscles from patients with IBM, showing early pathological changes. Similar significant differences of lower magnitude were also identified in CMT1A. (C) Fat-fraction maps of the right thigh in a healthy control and a patient with IBM. In the patient, fatty infiltration of muscles is greatest in the quadriceps (red region of interest), which also has a reduced CSA. The mean fat fraction and CSA can be combined to calculate the composite MRI metric, RMA. (D) RMA of quadriceps muscle showed significant correlation with knee extension strength in patients with IBM, CMT1A, and controls. Equivalent graphs of other movements are shown in the appendix. (E) Strong correlations were observed between mean thigh fat fraction and IBMFRS-LL (r=–0·64) for patients with IBM. FF=fat fraction. IBM=inclusion body myositis. CMT1A=Charcot-Marie-Tooth 1A. T2=transverse relaxation time. MTR=magnetisation transfer ratio. CSA=cross-sectional area. RMA=remaining muscle area. IBMFRS-LL=inclusion body myositis functional rating score lower limb.

Between Jan 19, 2010, and July 7, 2011, we recruited 20 patients with Charcot-Marie-Tooth disease 1A, 20 patients with inclusion body myositis, and 29 healthy controls allocated to one or both of the 20-participant control subgroups (figure 1). Age, sex, height, and weight were similar between the two patient groups and their respective matched controls (table 1). In both patient groups, baseline myometric muscle strength was significantly reduced in all muscle groups compared with their matched controls (table 1, appendix). At baseline, patients with inclusion body myositis had significantly higher all-muscle fat fraction and T2, and lower all-muscle MTR, at both thigh level and calf level than matched controls (table 1, figure 3). The all-muscle CSA in the inclusion body myositis group was lower than that of matched controls at thigh level but not at calf level (table 1). Patients in the Charcot-Marie-Tooth disease 1A group had significantly higher all-muscle fat fraction and T2 and lower MTR than matched controls at calf level but not at thigh level, whereas their all-muscle CSA was lower than for the controls at calf level but not at thigh level (table 1, figure 3).

Repeat assessments (table 2) were done after a mean of 12·4 months (SD 1·0). In the inclusion body myositis group, the MRC lower limb score, inclusion body myositis functional rating scale (IBMFRS), and overall myometric knee extension strength deteriorated significantly during follow-up. In the Charcot-Marie-Tooth disease 1A group, none of the clinical measures changed significantly. Although myometric ankle dorsiflexion strength showed a significant increase (p=0·049) compared with baseline, this was not significant (p=0·24) compared with the change in matched controls.

Quantitative MRI values for individual muscles are given in the appendix. Strong correlations were noted between the three quantitative MRI measures (fat fraction, MTR, and T2) of individual muscles (all r>0·89, p<0·0001, appendix).

**Figure 4:**
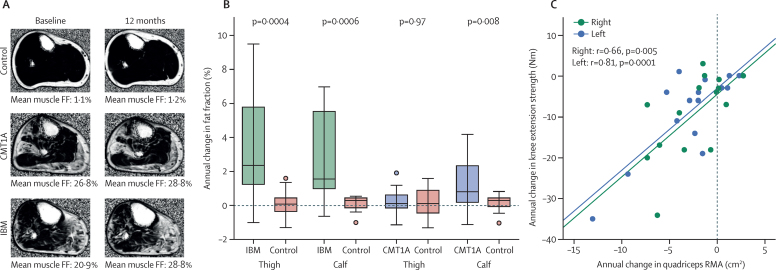
Longitudinal data Boxes represent median and IQR, whiskers show range and filled circles are outliers. (A) Fat-fraction maps of the right calf at baseline and after 1 year are shown for control participants and patients with CMT1A and IBM. Minimal change is seen in the mean overall fat fraction in the control, but an increase of 2·0% is shown in the patient with CMT1A and of 7·9% in the patient with IBM. (B) Group comparison against matched controls shows significant increases in overall mean fat fraction in patients with IBM at thigh and calf level and in patients with CMT1A at calf level. (C) Change in quadriceps RMA correlated with change in quadriceps strength over 12 months in patients with IBM for both left and right legs. No significant correlations were seen between 1-year changes in myometric and MRI measures in the CMT1A group. CMT1A=Charcot-Marie-Tooth 1A. IBM=inclusion body myositis. FF=fat fraction. RMA=remaining muscle area.

In patients with inclusion body myositis, at 12-month follow-up all-muscle thigh-level and calf-level fat fraction (figure 4), for both small and whole muscle ROIs, and calf muscle T2 had increased significantly from baseline, whereas calf muscle MTR significantly decreased (table 2). All-muscle CSA significantly decreased compared with baseline (p=0·01) at calf level but not at thigh level (p=0·08). In patients with Charcot-Marie-Tooth disease 1A, calf level all-muscle fat fraction increased significantly during the 12-month follow-up (figure 4), whereas T2, MTR, and muscle CSA did not change significantly (table 3), nor did any MRI measures at thigh level. In the control groups, no significant 12-month changes in fat fraction, T2, or MTR were reported at either level.

For the 29 control participants, the fat-fraction 95th percentiles were 4·8% for thigh muscles and 4·7% for calf-level muscles, defining the upper thresholds for intramuscular fat accumulation in these healthy controls. On regression analyses done separately for the inclusion body myositis and Charcot-Marie-Tooth disease 1A groups, combined with their respective controls, including only data from all individual patient and control muscles with fat fraction lower than these thresholds, T2 and MTR remained significantly dependent on fat fraction (appendix). However, the dependencies for control versus disease status were also significant in each case (appendix), with increased T2 (thigh 4·0 ms [SE 0·5], calf 3·5 ms [0·6]) and reduced MTR (thigh −1·5 pu [0·2], calf −1·1 pu [0·2]) independent of fat fraction in this regression model, in patients with inclusion body myositis versus matched controls. Smaller but significant fat-fraction-adjusted dependencies were also reported in patients with Charcot-Marie-Tooth disease 1A versus matched-controls, especially at calf level (T2 thigh 1·0 ms [0·3], calf 2·0 ms [0·3]; MTR thigh −0·3 pu [0·1], calf −0·7 pu [0·1]). The distribution of T2 and MTR values in muscles without substantial intramuscular fat accumulation in patients with inclusion body myositis is shown (figure 3).

In all groups, for all movements assessed, muscle strength correlated strongly with the corresponding muscle group total CSA (appendix). In regions where patients' fat fraction was significantly increased (inclusion body myositis thigh and calf level, Charcot-Marie-Tooth 1A calf level), whole muscle fat-fraction correlated negatively with muscle strength (appendix). The combined variable remaining muscle area correlated more strongly with strength than with either CSA or fat fraction separately (figure 3, appendix).

Significant correlations were also noted between summary clinical measures and all-muscle fat fraction measurements. In patients with inclusion body myositis, all-muscle thigh-level fat fraction correlated with disease duration (ρ=0·50, p=0·03, appendix), IBMFRS (ρ=–0·53, p=0·02), IBMFRS lower limb components (ρ=–0·64, p=0·002, figure 3), total MRC lower limb score (ρ=–0·60, p=0·005), and the physical function domain of the Short-Form 36 Quality of Life Score (SF36-PF; ρ=–0·60, p=0·007). Overall SF36 (ρ=–0·11, p=0·66) and age (ρ=0·14, p=0·55) were not significantly correlated with fat fraction. In patients with Charcot-Marie-Tooth 1A disease, all-muscle calf-level fat fraction correlated with disease duration (ρ=0·89, p<0·0001, appendix), age (ρ=0·84, p<0·0001), CMTES (ρ=0·63, p=0·003, appendix), lower limb motor component of the CMTES (ρ=0·77, p<0·0001), and reduced total MRC lower limb score (ρ=–0·76, p<0·0001). The correlation between all-muscle calf fat fraction and total SF36 score was not significant (ρ=–0·34, p=0·18); however, the correlation with the SF36-PF was significant (ρ=–0·63, p=0·007).

Correlations between the change in clinical variables and the change in MRI variables were not significant in the Charcot-Marie-Tooth group. However, in patients with inclusion body myositis, the change in myometric strength of knee extension correlated with change in several MRI variables, including quadriceps remaining muscle area (right r=0·66, p=0·005, left r=0·81, p=0·0001; figure 4).

The highest SRMs, greater than 1, were for whole muscle fat fraction at thigh and calf level, and T2 at calf level, whereas no clinical variables had an SRM greater than 1 (table 2). In the CMT group, significant 12-month change occurred only in whole muscle fat fraction at calf level, with an SRM of 0·83 (table 3).

## Discussion

In this study, quantitative MRI measures changed significantly during the 12-month follow-up in calf muscles of patients with Charcot-Marie-Tooth disease 1A and both thigh and calf muscles in patients with inclusion body myositis. MRI-measured fat fraction showed greater responsiveness (higher SRM) than clinical or myometric measures. Fat fraction correlated with strength and function. Even after adjustment for fat fraction, T2 was increased and MTR was reduced in muscles without substantial intramuscular fat accumulation in both patient groups compared with controls, suggesting sensitivity to early and potentially reversible changes in muscle water distribution. MRI therefore provides responsive outcome measures with validity suitable for application in future clinical trials of neuromuscular disorders.

The development of responsive outcome measures has proven especially difficult so far in neuromuscular disorders such as Charcot-Marie-Tooth 1A. The CMTNS was selected at the 136th European Neuromuscular Centre workshop^24^ as the most appropriate primary outcome measure for Charcot-Marie-Tooth 1A trials and was used in the ascorbic acid trials in adults.^6^, ^25^, ^26^ The SRM of the CMTNS and other outcome measures in the largest of these trials^6^ together with 5-year natural history data^4^ are shown in the appendix. On average, a 0·3 points per year increase in CMTNS is reported, resulting in minimal responsiveness over 2 years and small responsiveness over 5 years. Neurophysiology showed either no significant change from baseline^6^ or change no greater than seen in controls^4^ so seems unsuitable as an outcome measure. Ankle dorsiflexion showed mild responsiveness during 2 years using a custom built frame but no responsiveness during 5 years with hand-held myometry and in our study an apparent improvement in Charcot-Marie-Tooth 1A and controls was probably due to a learning effect. By marked contrast, in this study, calf-level MRI-quantified fat fraction showed large responsiveness (SRM=0·83) with a highly significant (p=0·002) increase in fat fraction at 12 months. This finding has important implications for future trial design. For a hypothetical Charcot-Marie-Tooth 1A treatment trial powered to detect a 50% reduction in disease progression during a 1-year period with 80% power at p<0·05 significance, the number of patients needed in active and placebo groups^27^ would be roughly 93 with calf muscle MRI-determined fat fraction as the primary outcome measure, as opposed to 7700 patients for the equivalent statistical power with CMTNS as the outcome. MRI-quantified calf muscle fat fraction is therefore the most responsive outcome measure proposed so far in Charcot-Marie-Tooth 1A.

Progression in inclusion body myositis is somewhat faster than in Charcot-Marie-Tooth 1A, with a 10-year median interval between symptom onset and significant disability.^28^ By contrast with the Charcot-Marie-Tooth 1A group, in the inclusion body myositis group, significant change during the 12-month follow-up was detected in IBMFRS, knee extension strength, MRC scores, myometry-measured knee extension, and many MRI measures. However, only MRI measure SRMs exceeded 1. Furthermore, quadriceps remaining muscle area correlated with change in knee extension strength (figure 4), showing for the first time a direct link between MRI-detected change and functional deficit. Thus, although MRI detected significant fat fraction progression during 12 months in both disease groups, in the more slowly progressive Charcot-Marie-Tooth 1A, calf-level MRI-measured fat fraction was the only measure to change significantly. In the more progressive inclusion body myositis, significant changes in several MRI, and some clinical and myometric, measures showed that the MRI indices were more responsive.

Additionally to responsiveness, outcome measures should show validity through correlation to relevant patient function. We showed strong clinical-MRI correlations for both overall participant and individual muscle measures. In both Charcot-Marie-Tooth 1A and inclusion body myositis, strong correlations exist between overall MRI measures and quality of life indices, functional or composite scales (IBMFRS and CMTES), and bedside strength examination, consistent with previous research.^9^, ^13^, ^29^, ^30^ At the individual muscle level, the intuitively expected correlation between CSA and strength in healthy controls was shown here, similar to a previous study.^31^ In both sets of patients, negative correlation between strength and fat fraction was noted, also shown previously in myotonic dystrophy for ankle dorsiflexion^9^ and in this study for both ankle and knee movements in both diseases. Muscle CSA and fat infiltration, when combined as remaining muscle area, correlated most strongly with strength. This might be superior to simple T1 and T2 measurements, which failed to show a correlation with strength in a study of patients with poliomyelitis.^32^ Thus, MRI provides indices of chronic muscle pathology that are highly correlated to muscle strength, but independent of participant effort or operator involvement, which lead to poor test-retest and interobserver reliability^33^ of direct muscle strength measures. MRI, therefore, provides a valid, reliable^16^ surrogate measure of muscle strength.

Because the T2 of fat greatly exceeds that of muscle water, and vice versa for MTR, T2 and MTR are affected by both fat fraction (exemplified by the strong inter-MRI variable correlations) and potentially independent early tissue water distribution changes. For this reason, T2 obtained in this manner is referred to as total T2 to distinguish from water-specific T2 obtained with methods eliminating the fat signal.^34^ Our data from muscles with fat fraction below the 95th percentile of the control range suggest that adjustment for T2 and MTR dependence on residual fat fraction shows significant differences between patient and control groups independent of fat fraction. These differences, greater for the inclusion body myositis group but also significant in Charcot-Marie-Tooth 1A, might represent early changes in muscle water distribution occurring before significant intramuscular fat accumulation. These changes might be reversible with therapy,^35^ and muscle T2 and MTR might thus provide useful biomarkers in clinical trials focused on early or active disease.

This study had several limitations. We analysed data from only single slices of thigh and calf blocks, with only small ROI for T2 and MTR sequences. This might have contributed to variation if muscle pathology was anatomically non-homogeneous. To ensure consistency in the volumes of tissue assessed longitudinally, slice positions were defined by measured distance from bony landmarks, a more reliable method than surface anatomy-based slice positioning,^36^ and follow-up ROIs were drawn with direct reference to the baseline ROI. T2 estimation by sampling two turbo-spin echo images is potentially less accurate than the Carr-Purcell-Meiboom-Gill multiple-spin-echo method.^37^ However, our approach provides time-efficient, wide-coverage quantification of T2 change using a method that can be implemented on standard MRI systems without specialist modification. Outcome measure SRMs derived from observational studies are applicable to interventional studies only when the intervention will affect the outcome measurement. For example, if an intervention had an effect on muscle that improved strength without an effect on muscle size or quantitative MRI variables, a functionally important benefit might have been missed. This association will need to be established in a disease-specific and intervention-specific manner.

Chronic intramuscular fat accumulation is common to a wide range of neuromuscular disorders. Although the precise molecular events responsible for intramuscular fat accumulation are not fully understood, one mechanistic study^38^ suggests that a range of different primary genetic muscle diseases, and potentially denervation, stimulate muscle precursor stems cells to differentiate into adipose cells and fibroblasts. That intramuscular fat accumulation seems to be a common pathway in many genetic and acquired neuromuscular disorders, underlines its potential usefulness as an outcome measure across neuromuscular disorders. As an example, we have shown here that the same types of change can be quantified in these two different muscle wasting diseases: an acquired late-onset progressive proximal and distal myopathy (inclusion body myositis) and an inherited childhood-onset slowly progressive distal predominant neuropathy (Charcot-Marie-Tooth 1A).

Although differences between diseases result in different distributions and degrees of muscle abnormalities, their presence, direction, and clinical correlations are consistent (panel 2), making these outcome measures potentially applicable across other neuromuscular disorders with lower limb weakness. Through selection of MRI variables targeted to disease and intervention, MRI outcome measures can be optimised to provide maximum responsiveness for a specific clinical trial. For example, in Charcot-Marie-Tooth 1A, in which the absence of significant MRI abnormalities at thigh level is a reflection of the length-dependent distribution of weakness, the optimum MRI protocol would include assessment of fat infiltration of calf muscles with a sequence such as 3-point Dixon. In inclusion body myositis, to assess whether an intervention reversed acute pathological processes, additional water-sensitive sequences such as T2 or MTR quantification should be included. In this study, T2 and MTR measurements showed similar responses, so the additional benefit of measuring both sequences remains unproven. For any specific neuromuscular disorders, understanding of basic disease mechanisms, disease distribution of the affected muscles, and treatment mechanisms will enable trial-specific selection of MRI outcome measures and appropriate anatomical imaging levels to provide optimum responsiveness.

In these representative neuromuscular disorders, the comprehensive MRC Centre MRI protocol provides outcome measures closely correlated to strength, function, and disease severity at baseline and longitudinally. In Charcot-Marie-Tooth 1A, responsiveness far exceeded that of existing outcome measures. This finding might allow progress in the design of adequately powered clinical trials in this gradually progressive but debilitating disease. In inclusion body myositis, T2 and MTR showed early changes in muscles before significant intramuscular fat accumulation, providing potential measures of early disease before irreversible changes have occurred. Together, the methods provide objective, non-invasive, valid, responsive outcome measures in inclusion body myositis and Charcot-Marie-Tooth 1A.

## Figures and Tables

**Table 1:** Baseline characteristics

		**Charcot-Marie-Tooth 1A group**	**Control group for Charcot-Marie-Tooth 1A disease**	**p value**	**Inclusion body myositis group**	**Control group for inclusion body myositis**	**p value**
**Demographics**							
Sex							
	Male	11	11	1	16	12	0·17
	Female	9	9	1	4	8	0·17
Age (years)		42·8 (13·9)	45·8 (14·2)	0·51	66·7 (8·9)	61·8 (10·3)	0·12
Height (cm)		167 (12)	171 (11)	0·34	175 (8)	171 (10)	0·28
Weight (kg)		70 (16)	75 (19)	0·44	84 (16)	77 (19)	0·22
Body-mass index		25·1 (4·6)	25·4 (4·9)	0·84	27·4 (4·0)	26·0 (4·6)	0·30
**Clinical parameters**							
Age of onset (years)		6·0 (4·4)	NA	NA	59·0 (8·5)	NA	NA
Disease duration (years)		35·8 (17·5)	NA	NA	7·7 (3·1)	NA	NA
CMTSS (0–12)*		3·1 (2·0)	NA	NA	NA	NA	NA
CMTES (0–28)*		8·0 (5·1)	NA	NA	NA	NA	NA
MRC-LL (0–110)*		95·4 (15·4)	NA	NA	93·4 (15·7)	NA	NA
SF36 (0–100%)		73·9% (15·2)	NA	NA	61·5% (15·3)	NA	NA
SF36-PF (0–100%)		65·3% (23·2)	NA	NA	39·5% (19·1)	NA	NA
IBMFRS (0–40)*		NA	NA	NA	27·6 (5·4)	NA	NA
**Myometric measures**							
Knee extension (Nm)		93·6 (44·1)	134·9 (43·5)	0·005	26·8 (26·0)	119·9 (43·0)	<0·0001
Knee flexion (Nm)		47·2 (20·1)	66·1 (20·4)	0·006	35·3 (19·9)	60·6 (20·1)	<0·0001
Ankle plantarflexion (Nm)		26·0 (14·3)	62·0 (19·1)	<0·0001	29·6 (15·7)	51·6 (18·4)	<0·0001
Ankle dorsiflexion (Nm)		10·8 (7·5)	30·0 (9·8)	<0·0001	13·2 (10·8)	28 (10·6)	<0·0001
**Thigh MRI**							
Fat fraction (%)		3·7% (6·8)	1·7% (1·3)	0·23	26·6% (15·5)	2·0% (1·2)	<0·0001
T2 (ms)		45·7 (9·1)	41·8 (3·1)	0·08	83·5 (17·7)	43·1 (2·5)	<0·0001
MTR (pu)		30·8 (3·0)	32·0 (0·8)	0·09	22·9 (5·0)	31·6 (0·7)	<0·0001
Fat fraction whole (%)		5·8% (8·5)	3·3% (1·8)	0·23	27·5% (15·7)	4·0% (1·6)	<0·0001
CSA (cm^2^)		201 (53)	219 (56)	0·31	169 (46)	212 (51)	0·009
**Calf MRI**							
Fat fraction (%)		15·8% (25·5)	1·6% (1·0)	0·02	18·0% (13·2)	2·0% (0·9)	<0·0001
T2 (ms)		59·7 (29·3)	40·2 (3·6)	0·005	71·1 (21·4)	41·9 (3·8)	<0·0001
MTR (pu)		26·1 (9·0)	32·1 (1·0)	0·007	23·7 (5·7)	31·6 (0·9)	<0·0001
Fat fraction whole (%)		15·5% (24·0)	2·7% (1·5)	0·03	19·2% (13·7)	3·5% (1·3)	<0·0001
CSA (cm^2^)		100 (26)	123 (27)	0·01	112 (26)	120 (31)	0·39

Data are presented mean (SD), unless otherwise indicated. MRI values are all-muscle region-of-interest means at the respective anatomical levels. For sex, the p value is for the Pearson χ^2^ test and for all other variables, the p value is for the two-tailed test. NA=not applicable. CMTSS=Charcot-Marie-Tooth symptom score. CMTES=Charcot-Marie-Tooth examination score. MRC-LL=Medical Research Council lower limb score. SF36=Short-Form 36 Quality of Life Score. PF=physical function domain. IBMFRS=inclusion body myositis functional rating scale. MTR=magnetisation transfer ratio. CSA=cross-sectional area. pu=percentage units

* Controls had no neuromuscular symptoms and were normal on neurological examination so all scored 0 on CMTES and CMTSS and attained the maximum score on IBMFRS (40) and MRC-LL (110).

**Table 2:** Change in all-muscle MRI and clinical measures between baseline and 12-month follow-up in patients with inclusion body myositis

	**Inclusion body myositis group (baseline *vs* 12-month follow-up)**	**Control group for inclusion body myositis (baseline *vs* 12-month follow-up)**	**Paired***t***test p value (12-month follow-up *vs* baseline in patient group)**	**Two-tailed p value (patient *vs* matched control over 12 month follow-up)**	**Standardised response mean**
**Overall clinical measures**					
MRC-LL*†	−3·4 (5·6; 0·6 to 6·2)	NA	0·02	NA	0·63
SF36 (%)	−1·1% (7·0; −2·7 to 4·8)	NA	0·55	NA	0·16
SF36-PF (%)	−4·1% (18·5; −13·9 to 5·8)	NA	0·39	NA	0·22
IBMFRS*†	−2·8 (2·9; 1·3 to 4·2)	NA	0·0008	NA	0·97
**Thigh myometry**					
Knee extension (Nm)	−6·0 (5·2; −8·4 to −3·6)	−4·2 (11·4; −9·5 to 1·1)	0·002	0·55	−1·15
Knee flexion (Nm)	−1·7 (4·3; −3·7 to 0·2)	2·9 (7·1; −0·4 to 6·2)	0·08	0·02	−0·40
**Calf myometry**					
Ankle plantarflexion (Nm)	−0·7 (3·9; −2·5 to 1·1)	5·6 (14·6; −1·2 to 12·4)	0·42	0·09	−0·19
Ankle dorsiflexion (Nm)	−0·4 (4·2; −2·4 to 1·5)	2·0; 5·3 (−0·4 to 4·4)	0·65	0·14	−0·10
**Thigh MRI**					
Fat fraction (%)†	3·3% (4·0; 1·4 to 5·2)	0·1% (0·4; −0·3 to 0·1)	0·005	0·001	0·83
T2 (ms)	2·6 (4·2; 0·5 to 4·7)	0·5 (1·6; −0·3 to 1·3)	0·03	0·07	0·62
MTR (pu)	−0·9 (1·6; −1·7 to 0·0)	0·0 (0·5; −0·2 to 0·2)	0·06	0·06	−0·54
Fat-fraction whole (%)†	3·3% (3·2; 1·8 to 4·9)	0·2% (0·8; −0·2 to 0·6)	0·0007	0·0004	1·06
CSA (% change)	−2·7% (7·9; −6·5 to 1·1)	0·2 (5·7; −2·5 to 2·9)	0·08	0·23	−0·34
**Calf MRI**					
Fat fraction (%)†	2·6% (2·7; 1·1 to 4·1)	0·0 (0·4; −0·2 to 0·2)	0·004	0·0007	0·97
T2 (ms)†	4·5 (3·7; 2·6 to 6·4)	0·0 (1·5; −0·7 to 0·7)	0·0005	<0·0001	1·21
MTR (pu)†	−0·7 (0·7; −1·1 to −0·3)	0·2 (0·8; −0·2 to 0·6)	0·004	0·003	−0·99
Fat-fraction whole (%)†	2·6% (2·4; 1·3 to 4·0)	0·1% (0·4; −0·1 to 0·3)	0·002	0·0006	1·07
CSA (% change)	−2·5% (3·9; −4·4 to −0·6)	0·1% (5·0; −2·3 to 2·5)	0·01	0·11	−0·63

Data are mean (SD; 95% CI), unless otherwise indicated. Fat-fraction whole and CSA are from whole muscle regions of interest, whereas fat fraction, T2, and MTR are from small regions of interest. NA=not applicable. MRC-LL=Medical Research Council lower limb score. SF36=Short-Form 36 Quality of Life Score. PF=physical function domain. IBMFRS=inclusion body myositis functional rating scale. MTR=magnetisation transfer ratio. CSA=cross-sectional area. pu=percentage units.

* Controls had no neuromuscular symptoms and were normal on neurological examination so attained the maximum score on IBMFRS (40) and MRC-LL (110), and they did not have quality of life assessments.

† Follow-up value is significantly different from baseline and change is significantly different from change in controls (both paired t test and two-tailed t test are significant).

**Table 3:** Change in all-muscle MRI and clinical measures between baseline and 12-month follow-up in patients with Charcot-Marie-Tooth 1A disease

	**Charcot-Marie-Tooth 1A group (baseline *vs* 12-month follow-up)**	**Control group for Charcot-Marie-Tooth 1A disease**	**Paired***t***test p value (12-month follow-up *vs* baseline in patient group)**	**Two-tailed p value (patient *vs* matched control over 12-month follow-up)**	**Standardised response mean**
**Overall clinical measures**					
MRC-LL*	−0·4 (3·8; −1·5 to 2·3)	NA	0·65	NA	−0·11
SF36 (%)	−2·5 (15·2; −5·3 to 10·3)	NA	0·51	NA	−0·16
SF36-PF (%)	−0·9 (12·3; −5·4 to 7·2)	NA	0·77	NA	−0·08
CMTES*	−0·3 (1·3; −0·9 to 0·4)	NA	0·37	NA	−0·23
**Thigh myometry**					
Knee extension (Nm)	1·0 (8·1; −2·8 to 4·8)	−5·2 (10·2; −9·9 to −0·4)	0·57	0·06	0·12
Knee flexion (Nm)	2·5 (6·8; −0·7 to 5·8)	1·7 (7·6; −1·7 to 5·2)	0·15	0·75	0·37
**Calf myometry**					
Ankle plantarflexion (Nm)	3·8 (7·6; 0·0 to 7·4)	2·1 (10·8; −2·9 to 7·1)	0·06	0·59	0·51
Ankle dorsiflexion (Nm)	2·1 (4·0; 0·2 to 4·0)	0·5 (3·8; −1·3 to 2·2)	0·05	0·24	0·51
**Thigh MRI**					
Fat fraction (%)	0·4 (1·0; −0·1 to 0·9)	0·0 (0·3; −0·1 to 0·1)	0·15	0·12	0·36
T2 (ms)	1·3 (1·5; 0·6 to 2·1)	0·6 (1·6; −0·2 to 1·4)	0·003	0·21	0·86
MTR (pu)	0·0 (0·7; −0·3 to 0·3)	−0·1 (0·3; −0·3 to 0·1)	0·96	0·55	−0·01
Fat-fraction whole (%)	0·2 (0·8; −0·2 to 0·6)	0·2 (0·8; −0·2 to 0·6)	0·38	0·97	0·22
CSA (% change)	−0·6 (5·3; −3·1 to 1·9)	−0·7 (7·2; −4·0 to 2·6)	0·62	0·96	−0·12
**Calf MRI**					
Fat fraction (%)	1·1 (2·4; 0·2 to 2·2)	0·0 (0·4; −0·2 to 0·2)	0·07	0·07	0·46
T2 (ms)	1·4 (2·6; 0·0 to 2·6)	0·3 (1·1; −0·2 to 0·9)	0·05	0·13	0·54
MTR (pu)	−0·2 (0·6; −0·5 to 0·1)	0·0 (0·7; −0·3 to 0·4)	0·30	0·28	−0·34
Fat-fraction whole (%)†	1·2 (1·5; 0·5 to 1·9)	0·2 (0·4; 0·0 to 0·4)	0·002	0·008	0·83
CSA (% change)	0·6 (6·6; −2·5 to 3·8)	0·0 (4·4; −2·0 to 2·0)	0·67	0·74	0·10

Data are mean (SD; 95% CI), unless otherwise indicated. Fat fraction whole and CSA are from whole muscle regions of interest, whereas fat fraction, T2, and MTR are from small regions of interest. NA=not applicable. MRC-LL=Medical Research Council lower limb score. SF36=Short-Form 36 Quality of Life Score. PF=physical function domain. CMTES=Charcot-Marie-Tooth 1A examination score. T2=transverse relaxation time. MTR=magnetisation transfer ratio. CSA=cross-sectional area.

* Controls had no neuromuscular symptoms and were normal on neurological examination so scored 0 on CMTES and attained the maximum score on MRC-LL (110), and they did not have quality of life tests.

† Follow-up value is significantly different from baseline and is significantly different from change in controls (both paired *t* test and two-tailed *t* test are significant).

## Panel 1 Technical terms

**Statistical terms**

Responsiveness: the ability of a measure to change over a prespecified timeframe (internal responsiveness). External responsiveness represents the extent to which a change in a measure relates to corresponding change in a reference measure of clinical or health status.

Standardised response mean (SRM): a measure of outcome measure responsiveness calculated from natural history or placebo data by dividing the mean change by the standard deviation. An SRM of less than 0·2 is thought to have minimal responsiveness and an SRM of more than 0·8 is deemed to have large responsiveness. In addition to having a high SRM, the measurement must also be expected to respond to the planned intervention.

Lehr's formula:

$$\text{n}=\frac{16}{{\left(E\cdot SRM\right)}^{2}}$$

A formula to estimate the number of participants in each group for a study with 80% power to detect an intergroup difference with p<0·05 significance. It is based on SRM and effect size in terms of reduction in progression versus placebo (E). For example, if a clinically significant benefit is regarded as a 50% reduction in disease progression (E=0·5), use of an outcome measure with minimal responsiveness (SRM=0·2), would require 1600 participants in each study group, whereas an outcome measure with large responsiveness (SRM=0·8) would require 100 participants in each group.

Validity: measured by comparing the results of a measurement to the results of a gold standard test. For an outcome measure, validity here refers to correspondence with clinical measures of patient function, considered along with mortality as possible “true outcome measures” in a clinical trial.

**MRI terms**

Fat fraction: the proportion of water (W) to fat (F) signal originating in the measurement volume reported as a percentage.

$$\text{Fat}\;\text{fraction}=\frac{\text{F}}{\left(\text{F}+\text{W}\right)}\times 100\%$$

3-point-Dixon fat water separation: an MRI technique yielding separate fat-only and water-only images

Remaining muscle area: defined here as the cross-sectional area of muscle tissue within the muscle fascial boundary (CSA) not replaced by fat.

Transverse relaxation time (T2): a key source of MRI contrast representing changes in molecular mobility, commonly interpreted in terms of tissue water or lipid distribution changes.

Magnetisation transfer ratio (MTR): an MRI measure being the signal intensity ratio between a control image and one made sensitive to changes in the relative population of free water in tissue and the rate of magnetisation exchange with other proton pools.

## Panel 2 Research in context

**Systematic review**

We searched PubMed for reports published before July 1, 2015, with the MeSH terms “magnetic resonance imaging” and “Charcot-Marie-Tooth disease”, “myositis, inclusion body”, or “neuromuscular diseases”, with no language restrictions. We restricted our search to reports in human participants. Five articles were identified reporting skeletal muscle MRI in inclusion body myositis, but none included quantitative MRI sequences and none included longitudinal data. 12 articles were identified reporting skeletal muscle MRI in Charcot-Marie-Tooth 1A. Only two of these^14^, ^39^ used quantitative MRI sequences but did not include longitudinal data. Of the ten studies reporting qualitative MRI in Charcot-Marie-Tooth 1A, one study included a longitudinal scan on a single patient,^40^ whereas one study reported qualitative MRI imaging in 14 patients at a 2 year interval but identified no progression.^41^ Taking into account all neuromuscular disorders, published quantitative MRI studies have quantified fat fraction,^9^, ^10^, ^11^, ^12^, ^13^ T2,^9^, ^11^, ^29^, ^30^, ^42^, ^43^ and magnetisation transfer ratio,^14^, ^44^ in disorders that are myopathic,^9^, ^10^, ^11^, ^12^, ^13^, ^29^, ^30^, ^42^, ^43^, ^44^ neuropathic,^10^, ^14^ inherited,^9^, ^10^, ^11^, ^12^, ^13^, ^14^, ^29^, ^30^, ^42^, ^44^ or acquired,^14^, ^43^ at thigh level^10^, ^11^, ^13^, ^30^, ^42^, ^43^ or calf level,^9^, ^11^, ^14^, ^29^, ^42^, ^44^ in one muscle^9^, ^10^, ^29^ or many muscles,^11^, ^12^, ^13^, ^14^, ^30^, ^42^, ^43^, ^44^ and with controls^9^, ^11^, ^14^, ^29^, ^42^, ^43^, ^44^ or without controls.^10^, ^12^, ^13^, ^30^ Few studies in patients with neuromuscular disorders have been published so far with longitudinal quantitative MRI data.^13^, ^15^, ^45^, ^46^ Observational studies have shown longitudinal change in muscle fat fraction in patients with limb girdle muscular dystrophy type 2I,^13^ and longitudinal change in muscle T2 in Duchenne muscular dystrophy.^15^ Studies in Duchenne have used MRI longitudinally after steroid initiation and showed a reduction in T2^45^ or a variable effect on T2.^46^

**Interpretation**

Accurately and reliably monitoring disease progression is an important barrier to successful experimental clinical trials to test new therapies for muscle-wasting neuromuscular diseases. We report longitudinal data in patients with one of two representative neuromuscular disorders and healthy controls. We have systematically investigated quantitative MRI biomarkers and have shown they can reliably and accurately track disease progression. In patients with Charcot-Marie-Tooth 1A, calf muscle fat fraction showed high responsiveness, exceeding all clinical measures. Abnormalities in T2 and MTR, measures sensitive to muscle water accumulation, were seen in muscles before significant fat infiltration. We have also shown correlations with important measures of patient function including strength, functional scores, and quality of life measures. The biomarkers we selected detect and quantify water and fat accumulation within muscle, which both occur across a range of different muscle-wasting diseases. Therefore these quantitative MRI methods might be applicable to other muscle-wasting neuromuscular diseases.
