# Supplementary material for: MRI biomarker assessment of neuromuscular disease progression: a prospective observational cohort study
Source: Lancet Neurol. 2016 Jan;15(1):65–77. doi: 10.1016/S1474-4422(15)00242-2 (PMC4672173; doi:10.1016/S1474-4422(15)00242-2)
Supplement: Supplementary appendix [file mmc1.pdf]

THE LANCET Neurology

Supplementary webappendix

This webappendix formed part of the original submission and has been peer reviewed. We post it as supplied by the authors.

Supplement to: Morrow JM, Sinclair CDJ, Fischmann A, et al. MRI biomarker assessment of neuromuscular disease progression: a prospective observational cohort study. *Lancet Neurol* 2015; published online Nov 5. http://dx.doi.org/10.1016/S1474-4422(15)00242-2.

WEB EXTRA MATERIAL

Supplementary methods:

Disease overview

Inclusion body myositis (IBM) is an acquired inflammatory myopathy with onset usually over 50 years of age with a male predominance and the quadriceps being the predominant muscle affected in the lower limbs. There are currently no treatments with proven efficacy despite numerous trials.[1] Charcot-Marie-Tooth disease (CMT) is an umbrella term for the hereditary motor and sensory neuropathies with a combined prevalence of around 1:2500 and over 70 causative genes identified. The most common type is CMT1A representing 50% of all CMT with progressive distal weakness from childhood.[2] Recent trials have been hampered by insensitive outcome measures.[3]

Exclusion criteria: (all subjects)

- Concomitant neuropathy/myopathy
- Very advanced disease state that precludes travelling
- Severe cardiovascular, renal, or other end-stage-organ-disease states or any other major comorbidities (e.g. any active malignancy, definite cognitive impairment, psychiatric disease, heart or lung failure, orthopedic or rheumatologic disorders)
- Pregnancy and active nursing (breast feeding)
- Inadequate contraception in women of child bearing age.
- Contra-indication to MRI (metallic fragments, clips or devices in the brain, eye, spinal canal, etc; magnetically activated implanted devices, such as cardiac pacemakers, insulin pumps, neurostimulators and cochlear implants; claustrophobia)
- Existing radiculopathy or lower back pain
- Surgery to feet within 12 months of beginning of study or during the study

Clinical Strength Grading (MRC Scale)

The following movements were assessed:

- Neck: flexion and extension
- Upper limbs: shoulder abduction, elbow extension, elbow flexion, wrist extension, wrist flexion, finger extension, forefinger abduction, little finger abduction, thumb abduction, long finger flexors, short finger flexors
- Lower limbs: hip flexion, hip extension, hip abduction, hip adduction, knee flexion, knee extension, ankle dorsiflexion, ankle plantarflexion, ankle eversion, ankle inversion, big toe extension

Muscle strength was assessed using a modified MRC scale[5] with 5: normal strength, 5-: barely detectable weakness, 4+: gravity and moderate to maximal resistance, 4: Gravity and moderate resistance, 4-: Gravity and minimal resistance, 3: Full range of motion against gravity only, 2: Movement when gravity is eliminated, 1: Flicker of movement seen or felt, 0: No movement. Upper limb/neck and lower muscle scores were summed to obtain a total upper limb/neck and total lower limb score for each subject. For this purpose 5- was scored as 4.75, 4+ as 4.25 and 4- as 3.75. The maximum score obtainable was 120 upper limb/neck and 110 for lower limb.

Myometry protocol

All but two initial assessments and all follow up assessments were performed by a single neurologist. Myometry was performed according to the table below. Knee extension, knee flexion, ankle dorsiflexion, and ankle plantarflexion were assessed bilaterally using both isometric and isokinetic protocols and the maximum torque in Nm recorded for analysis. Isometric assessments consisted of four attempts of 3 seconds duration with 10s interval of which the best attempt was selected. For the isokinetic assessments, following a practice run and 10s interval, three successive movements through full range were performed and the highest value obtained selected. The machine setup was recording at first visit using the included software, which was then retrieved to allow identical set-up on repeat testing. A single observer performed over 90% of assessments. All measurements are stored within the HUMAC NORM system, and were checked for artefacts before being exported into IBM-SPSS for analsysis. An additional overall strength for each movement was calculated by taking the mean of all assessment methods. The Myometry assessment either occurred following the MRI or with at least a 60 minute gap if preceding to minimise any potential MRI effects secondary to physical activity.

Joint	Type	Movement	Angle
Knee (right then left)	Isometric	Extension°	45°
		Extension	90°
		Flexion	45°
		Flexion	90°
	Isokinetic	Extension/Flexion	60°/s
		Extension/Flexion	120°/s
Ankle (left then right)	Isometric	Plantarflexion	10°
		Dorsiflexion	10°
	Isokinetic	Plantarflexion/Dorsiflexion	60°/s

MRI Protocol

Selection of musculature
Lower limb muscles were chosen as the region for study as they are a key site of pathology in both CMT1A (lower calf)[2] and IBM (quadriceps)[1] and weakness is these areas is a key cause of disability in these patient groups. Furthermore lower limb imaging is practical compared with dedicated upper limb image: both limbs may be imaged simultaneously lowering scanning times and lower limb imaging is in our experience more comfortable for participants.

Block positioning
Imaging was performed using a multi-channel peripheral angiography coil (Siemens 'PA Matrix') and 'spine matrix' coil elements supplemented with a body surface coil for proximal thighs. Before scanning the distance between the anterior superior iliac spine and the superior border of the patella was measured and thigh-level imaging volumes were centred one third of this distance above the patella superior border. Similarly calf-level imaging volumes were centred below the tibial tuberosity by one quarter of the total distance from the tibial tuberosity to the lateral malleolus. The derived distances were recorded and used for follow up imaging.

Field of View (FOV)
Both limbs were scanned within the FOV. Axial FOVs were 41x20.5cm (thigh) and 40x18.8cm (calf)

3-Point Dixon Fat-Fraction (FF) Quantification
For Dixon FF measurements,[6] three 2D gradient-echo acquisitions were performed with echo-times (TE1/TE2/TE3=3.45/4.60/5.75ms, TR=100ms, flip angle=10°, bandwidth 420Hz/pixel, NEX=4, 10 x 10mm slices with 10mm gap, 512x256matrix (thigh), 512x240matrix calf, iPat=2). Phase unwrapping was performed using PRELUDE (FSL, FMRIB, Oxford)[7] and after fat (F) and water (W) image decomposition, FF calculated as FF = 100% x F/(F+W).

The TE=3.45ms image was used for the region of interest (ROI) placement and as a reference for inter-method image interpolation and registration (calf) using FLIRT (FSL, FMRIB, Oxford), such that that the same ROIs could be applied to extract data from all maps.

T_2-Relaxometry
Dual-contrast turbo-spin-echo (TSE) images (6500/13/52ms or 6500/16/56ms; 10x10mm slices with 10mm gap, iPat=2, BW=444Hx/pixel, refocusing flip angle 180º, NEX=1, 6/8 *k*-space sampling, 256x128matirx (thigh), 256x120matrix (calf)) were acquired. Pseudo-T_2 was calculated from the respective pixel intensities I_{TE1} and I_{TE2} from the TE_1 and TE_2 images as $T_2= \frac{TE_2 - TE_1}{\ln(I_{TE1}/I_{TE2})}$. The different echo times were the result of altered constraints following a routine scanner software upgrade which occurred after 54 baseline and 6 follow-up scans had been completed. Analysing control values pre- and post-upgrade suggested a systematic bias between pre- and post-upgrade T2 values. By comparing the observed relationship between FF and T2 measurements pre- and post-software upgrade a correction equation was determined separately for thigh muscles (corrected T2 = 1.0606 x post-upgrade T2 + 1.1522) and calf muscles (corrected T2 = 1.0933 x post-upgrade T2 – 0.0245). These corrections were applied to all post-upgrade T2 measurements to ensure pre- and post-upgrade T2 measurements were comparable. Parameters for the other quantitative sequences were not affected by this upgrade, and analysis of the control values pre- and post-upgrade indicated no systematic bias in these values was introduced by the software upgrade.

B_1 Mapping
Separate TSE images (TR/TE=7000/11ms, 128x64 matrix, 40 contiguous 10mm slices, BW=429Hx/pixel, 1/2 *k*-space sampling) yielded image intensities V_1 and V_2 acquired with nominal excitation α_1 and α_2 of 60° and 120°. B_1 deviation was mapped according to $B_{1Dev} = \arccos(V_2/2V_1)/\alpha_1$.[8]

Magnetization Transfer Ratio (MTR) Imaging
MTRs were calculated from two 3D-FLASH images with (M_1) and without (M_0) an MT pre-pulse (500° amplitude, 1200Hz offset, 10ms duration) (TR/TE=65/3ms or 68/3ms, α=10º, BW=440Hz/pixel, NEX=1, 6/8 *k*-space sampling, iPat=2, 40x5mm longitudinal phase encoding partitions, 256x128matrix (thigh), 256x120matrix (calf)) according to MTR = $(M_0-M_1)/M_0$ x 100 percentage units (p.u.). Percentage units were used by convention to avoid ambiguity with fractional change expressed as a percentage.[9] MTR maps were RF-inhomogeneity corrected using the B_1 maps according using a mean-over-all-subjects B_1 inhomogeneity correction factor of k = 0.0085.[10]

Analysis Slice Selection
For the baseline scan the fifth most superior slice was used in the thigh and the sixth slice in the calf unless all muscles were not visible in which case an adjacent slice was selected. The ROI were drawn on the follow up acquisition on the slice closest to that used for the first scan, determined on the basis of measured distance from bony landmarks (tibial plateau or tip of the fibular head) identified on the 3D-FLASH images.
After extraction, all data were cross-checked for outliers and any errors rectified.

Data analysis

Whole muscle ROIs were defined to encompass the entire muscle cross sectional area (CSA) to the fascia whilst "small" ROIs were defined in a consistent anatomical location within each muscle to avoid contamination with fascia or vessels and to allow for minor anatomical movement between acquisitions (figure 2A). Left and right limb ROIs were defined for all muscles at these levels: rectus femoris, vastus lateralis, vastus intermedius, vastus medialis, semimembranosus, semitendinosus, biceps femoris, adductor magnus, sartorius, gracilis, tibialis anterior, peroneus longus, lateral gastrocnemius, medial gastrocnemius, soleus and tibialis posterior muscles.

Minor adjustments to small ROI were made where imperfect registration meant ROI were no longer wholly within the target muscle. Whole muscle ROIs were transferred to the inherently co-registered FF maps only. The whole muscle ROIs from the unprocessed Dixon acquisition were not used for T2 and MTR analysis to avoid ROI contamination with non-muscle tissue, a particular problem for these measures, at the region boundaries due to minor subject movement between acquisitions. ROIs including areas of gross artefact were excluded from the analysis. Of 248 total maps, 5 fat fraction maps (2%), 2 T2 maps (1%) and 14 MTR maps (5%) were excluded due to technical issues or widespread artefact. Artefact in the anterior right thigh of the MTR maps limited analysis in this region as reported in the application of this protocol to healthy volunteers.[4] This is demonstrated in the number of each ROI analysed in figure e1.

Statistical methods

If the follow-up duration differed from 12 months the magnitude of change was converted to an annualised value. Outcome measure 12 month SRMs were calculated for each measure as the mean change between baseline and follow-up divided by the standard deviation of that change, and categorised by magnitude according to Cohen's suggestion: <0·2 minimal responsiveness; 0·2-0·5 small responsiveness; 0·5-0·8 moderate responsiveness; >0·8 large responsiveness.

Table e1: Baseline myometry statistics by group

Movement	Type	Side/angle	CMT	Control (CMT)	IBM	Control (IBM
Knee Extension	Isometric	Right at 45°	111.1 ± 48.9 (24-209)	155.6 ± 47.3 (73-259)	35.4 ± 38.4 (0-146)	138.6 ± 60 (18-259)
		Left at 45°	109.7 ± 47.6 (38-217)	152.2 ± 45.5 (73-231)	33.5 ± 38.3 (0-145)	138.9 ± 48.7 (62-218)
		Right at 90°	112.2 ± 58.4 (45-290)	159.3 ± 62.6 (84-292)	29.2 ± 23.9 (2-91)	136.6 ± 55.4 (18-252)
		Left at 90°	106.6 ± 60.8 (43-277)	142 ± 50.5 (72-247)	28.3 ± 26.5 (0-94)	128.5 ± 43.3 (72-216)
	Isokinetic	Right at 60°/s	87 ± 45 (12-184)	133.5 ± 49.1 (49-239)	25 ± 22 (0-87)	116.8 ± 45.4 (18-190)
		Left at 60°/s	87.4 ± 42.1 (23-165)	127.6 ± 40 (52-202)	24.2 ± 24.4 (0-94)	115.7 ± 38 (52-174)
		Right at 120°/s	70 ± 32.5 (27-138)	106.9 ± 40.8 (41-206)	20.2 ± 18.3 (0-71)	93 ± 38.5 (13-170)
		Left at 120°/s	68.6 ± 35.3 (26-138)	102.5 ± 35.2 (45-165)	19 ± 19.7 (0-80)	91.4 ± 32.8 (45-145)
Knee Flexion	Isometric	Right at 45°	65.8 ± 26.9 (33-115)	86.6 ± 28.9 (53-141)	49.6 ± 27.4 (2-110)	80 ± 29.1 (33-140)
		Left at 45°	66.3 ± 27.5 (33-113)	81.8 ± 22.9 (35-132)	48.8 ± 25.5 (2-106)	74.9 ± 27.7 (35-132)
		Right at 90°	47.9 ± 20.4 (14-92)	64.7 ± 27.1 (31-127)	28.9 ± 18.1 (0-68)	54.7 ± 18.9 (26-106)
		Left at 90°	45.4 ± 20.9 (15-83)	57.8 ± 24.3 (28-110)	31.3 ± 20.9 (0-77)	49.3 ± 18.3 (24-95)
	Isokinetic	Right at 60°/s	46.6 ± 18.8 (9-81)	70 ± 21.7 (28-123)	34.9 ± 21.4 (0-81)	64.5 ± 21 (27-99)
		Left at 60°/s	45.1 ± 20 (8-85)	62.9 ± 17.3 (20-85)	37 ± 21.5 (0-80)	62.4 ± 22.7 (20-104)
		Right at 120°/s	35.1 ± 16.8 (14-72)	55.5 ± 19.7 (22-102)	26.1 ± 17.7 (0-72)	50.8 ± 17.8 (22-87)
		Left at 120°/s	34.5 ± 18.6 (12-75)	50 ± 14.9 (20-77)	25.9 ± 17 (0-57)	48.5 ± 18.2 (20-84)
Ankle Plantar-flexion	Isometric	Right at 10°	33.7 ± 19.6 (1-64)	66.6 ± 20.8 (35-106)	34.4 ± 18.7 (3-64)	56.2 ± 19.1 (28-92)
		Left at 10°	32.7 ± 19.8 (0-64)	65.1 ± 19.5 (33-108)	33.6 ± 16.7 (4-60)	57.2 ± 23.7 (33-110)
	Isokinetic	Right at 60°/s	18.7 ± 12.8 (5-58)	57.7 ± 21.6 (24-107)	24.1 ± 14.3 (0-54)	46.1 ± 17 (22-75)
		Left at 60°/s	19 ± 12.4 (4-53)	58.6 ± 21.7 (22-115)	29.2 ± 17.2 (7-71)	46.9 ± 20.5 (8-79)
Ankle Dorsi-flexion	Isometric	Right at 10°	9.7 ± 8.1 (0-31)	35.7 ± 13.7 (16-60)	15 ± 12.9 (0-38)	33 ± 14.9 (7-60)
		Left at 10°	10 ± 7.8 (0-27)	34.5 ± 13.1 (12-58)	15.4 ± 12.5 (0-38)	32.6 ± 14.2 (8-58)
	Isokinetic	Right at 60°/s	11.9 ± 8.4 (0-30)	25.4 ± 8.5 (12-42)	11.8 ± 12.5 (0-50)	23.3 ± 9.2 (9-42)
		Left at 60°/s	11.9 ± 11 (0-43)	24.5 ± 8.7 (7-37)	11.8 ± 8.3 (0-26)	23.2 ± 9 (7-35)
Ankle Inversion	Isometric	Right at 0°	15.6 ± 10 (0-37)	20.4 ± 7.1 (9-33)	11.9 ± 7.3 (0-27)	18.1 ± 6.4 (9-33)
		Left at 0°	14.8 ± 10.4 (0-45)	20.1 ± 7.1 (7-30)	11.5 ± 6.7 (0.15-26)	17.7 ± 6.7 (7-30)
	Isokinetic	Right at 60°/s	16 ± 9.9 (0-38)	25.4 ± 7.9 (11-39)	14.9 ± 9.6 (3-34)	22.7 ± 7.8 (9-34)
		Left at 60°/s	14.3 ± 9.5 (3-35)	25.6 ± 8.9 (11-43)	17.1 ± 10.2 (3-35)	21.8 ± 8.6 (11-39)
Ankle Eversion	Isometric	Right at 0°	7.1 ± 4.1 (1-14)	22 ± 7.6 (9-37)	9.6 ± 5.9 (0-22)	19 ± 7.6 (9-34)
		Left at 0°	7.7 ± 4.8 (0-18)	21.4 ± 7.1 (11-34)	10.2 ± 6.5 (0-20)	17.5 ± 6.9 (8-31)
	Isokinetic	Right at 60°/s	8.4 ± 4 (1-15)	17.3 ± 5.6 (9-28)	9.2 ± 5 (0-20)	15.1 ± 5.6 (8-23)
		Left at 60°/s	7.8 ± 3.1 (3-14)	17.4 ± 5.8 (9-30)	9.3 ± 5.8 (0-24)	16.2 ± 7.1 (8-33)

Data are mean ± standard deviation (range). Isometric values are the peak torque at the fixed angle listed whilst isokinetic values are the peak torque at the fixed speed noted. Both CMT1A patients and IBM patients have significantly ($p<0.01$ for all) reduced strength than their matched control groups for all measurements.

Table e2: baseline individual muscle MRI values in CMT and matched controls

	CMT				Control (CMT)			
Muscle	FF (%)	T2 (ms)	MTR (p.u.)	FFw (%)	FF (%)	T2 (ms)	MTR (p.u.)	FFw (%)
Right Rectus Femoris	3.6 ± 12.1 (20)	46.8 ± 13.7 (16)	28.9 ± 9.3 (5)	6.3 ± 13 (20)	0.7 ± 1 (20)	40.8 ± 5.5 (17)	33.3 ± 1.9 (3)	2.7 ± 1.9 (19)
Right Vastus Medialis	2 ± 2.9 (20)	43.8 ± 7.8 (20)	31.6 ± 2.2 (10)	4.4 ± 8.1 (20)	1 ± 0.8 (20)	40.9 ± 3.9 (19)	32.6 ± 1.9 (8)	2.2 ± 1.1 (19)
Right Vastus Intermedius	3 ± 5.8 (20)	44.6 ± 11.5 (20)	30.3 ± 4.8 (14)	4.3 ± 10.4 (20)	1 ± 1.1 (20)	38.4 ± 2.8 (20)	33.2 ± 1.8 (14)	2 ± 1.6 (19)
Right Vastus Lateralis	5.4 ± 19.1 (20)	49.6 ± 26.2 (19)	31 ± 6.6 (20)	6.2 ± 14 (20)	1.2 ± 0.9 (20)	42.2 ± 4.8 (18)	32.5 ± 1.3 (19)	2.6 ± 1.3 (19)
Right Semimembranosus	3.5 ± 4.5 (20)	48.5 ± 8.8 (19)	30.5 ± 2.9 (20)	6.7 ± 5.5 (20)	2.5 ± 3.1 (20)	44.9 ± 6.4 (20)	31.4 ± 1.6 (18)	4.6 ± 2.9 (19)
Right Semitendinosus	2.3 ± 2.7 (20)	47.4 ± 8.3 (20)	31.2 ± 2.8 (20)	6.3 ± 5.3 (20)	1.1 ± 0.7 (20)	43.3 ± 4 (20)	32.1 ± 1.1 (18)	3.6 ± 2.7 (19)
Right Biceps Femoris	2.7 ± 3.5 (20)	47.8 ± 7.4 (19)	30.5 ± 2.6 (20)	4.6 ± 4.7 (20)	2 ± 1.4 (20)	46.5 ± 5 (18)	31.2 ± 1.3 (18)	3.7 ± 2.4 (19)
Right Adductor Magnus	2.3 ± 4 (20)	45 ± 7.3 (20)	31.9 ± 2.2 (20)	5.1 ± 7.3 (20)	2 ± 3 (20)	43 ± 5.6 (20)	32.1 ± 1.6 (18)	3.8 ± 3.4 (19)
Right Sartorius	5.9 ± 10.6 (20)	48.3 ± 11.1 (20)	27.7 ± 5.4 (13)	9.5 ± 8.6 (20)	3.6 ± 3.8 (20)	43.6 ± 6.1 (19)	31.9 ± 1.8 (8)	5.9 ± 2.8 (19)
Right Gracilis	3.1 ± 4.6 (19)	44 ± 7.5 (20)	31.8 ± 3.2 (19)	6.8 ± 5.7 (20)	1.8 ± 1.7 (20)	39.5 ± 3.5 (20)	32.8 ± 1.4 (17)	4.1 ± 2.3 (18)
Left Rectus Femoris	2.7 ± 8.2 (20)	45.4 ± 17.1 (20)	31.6 ± 4.6 (16)	6 ± 11.4 (20)	1.1 ± 1.6 (20)	38.4 ± 4.8 (18)	32.8 ± 1.9 (18)	3 ± 2.3 (19)
Left Vastus Medialis	2.7 ± 5.4 (20)	44.3 ± 9.2 (20)	31.5 ± 3.4 (19)	5.3 ± 9.3 (20)	1.2 ± 1.3 (20)	42 ± 4.7 (20)	32.3 ± 1.2 (18)	2.7 ± 1.4 (19)
Left Vastus Intermedius	8.2 ± 21.7 (20)	51.8 ± 29.6 (20)	29 ± 7.7 (19)	6.1 ± 12.6 (20)	1.3 ± 1 (20)	42.3 ± 5.5 (19)	31.7 ± 1.4 (19)	2 ± 1.3 (19)
Left Vastus Lateralis	4.8 ± 15.1 (20)	44.5 ± 18.7 (20)	30.8 ± 5.6 (19)	7.2 ± 15.1 (20)	1.6 ± 1.2 (20)	40.6 ± 3 (19)	32.1 ± 1.2 (19)	3.4 ± 1.7 (19)
Left Semimembranosus	3 ± 1.8 (20)	43.5 ± 4.4 (20)	29.6 ± 2.4 (16)	6.9 ± 5.5 (20)	2.3 ± 1.8 (19)	42 ± 5.1 (20)	30.9 ± 1.1 (17)	4.2 ± 2.6 (17)
Left Semitendinosus	2.6 ± 3.1 (20)	42.7 ± 5.6 (20)	31.1 ± 2.6 (18)	6.4 ± 5.4 (20)	1.4 ± 1.2 (19)	40.8 ± 4.3 (20)	31.9 ± 1 (18)	4.1 ± 2.3 (17)
Left Biceps Femoris	3 ± 4.2 (20)	44.8 ± 7.4 (20)	30.7 ± 3.2 (19)	6.1 ± 5.7 (19)	2.6 ± 2.2 (20)	43.5 ± 6.4 (20)	31.8 ± 1.4 (19)	4.8 ± 2.8 (19)
Left Adductor Magnus	2 ± 2.2 (20)	41.9 ± 4.2 (20)	30.7 ± 2.1 (18)	6.2 ± 8.9 (19)	2 ± 2.3 (20)	42.4 ± 5.2 (20)	31.6 ± 1.3 (18)	3.9 ± 3.3 (19)
Left Sartorius	5.5 ± 9.9 (20)	47.4 ± 13.2 (20)	30.2 ± 4.6 (17)	9.1 ± 9.1 (20)	2.7 ± 2.9 (20)	42 ± 6 (20)	31.8 ± 2.3 (16)	6.7 ± 3.5 (19)
Left Gracilis	5 ± 11.5 (20)	43.7 ± 13.4 (20)	30.4 ± 4.2 (18)	7.5 ± 9.7 (20)	1.8 ± 1.6 (20)	38.1 ± 3.8 (20)	32.4 ± 1.4 (18)	5 ± 2 (19)
Right Tibialis Anterior	12.6 ± 23.6 (20)	57.2 ± 29.7 (20)	23.4 ± 8.4 (16)	14 ± 21.2 (20)	0.7 ± 0.7 (19)	36.9 ± 2.3 (20)	30.5 ± 1.4 (17)	1.5 ± 0.9 (19)
Right Peroneus Longus	21.9 ± 30.5 (20)	66.4 ± 36.1 (20)	24.2 ± 10.9 (20)	24.4 ± 28.6 (20)	1.6 ± 1.5 (19)	40.6 ± 5.1 (19)	31.9 ± 1.6 (18)	3.1 ± 2.4 (19)
Right Lateral Gastrocnemius	15.1 ± 29 (20)	60.2 ± 34 (20)	25.8 ± 10.2 (20)	15.8 ± 27.3 (20)	1.3 ± 0.9 (19)	40.4 ± 4.7 (20)	32.1 ± 0.9 (17)	2.5 ± 1.5 (19)
Right Medial Gastrocnemius	18.2 ± 29.1 (20)	61.5 ± 30.8 (20)	25.3 ± 10.2 (19)	18.6 ± 28.1 (20)	1.5 ± 0.9 (18)	40.2 ± 3.5 (20)	32.7 ± 1.3 (17)	2.5 ± 1.2 (18)
Right Soleus	13 ± 25.8 (20)	56.4 ± 29.6 (20)	27.6 ± 9.4 (19)	12.6 ± 23.7 (20)	2.7 ± 2.9 (19)	43 ± 6.5 (20)	32 ± 1.6 (18)	3 ± 2.7 (19)
Right Tibialis Posterior	12.4 ± 25.9 (20)	56.5 ± 34.2 (19)	27.7 ± 9.5 (20)	12.4 ± 22.6 (20)	1 ± 0.9 (19)	38.9 ± 2.8 (20)	32.9 ± 1.1 (18)	1.5 ± 1 (19)
Left Tibialis Anterior	16.8 ± 28.4 (19)	64.5 ± 35.5 (19)	25.4 ± 9.4 (20)	15.7 ± 22.2 (19)	0.9 ± 0.7 (19)	40.3 ± 5.9 (20)	32 ± 1.4 (18)	2 ± 1 (19)
Left Peroneus Longus	18.9 ± 30 (20)	61.1 ± 32.5 (19)	25.8 ± 10.4 (20)	22.4 ± 27.8 (20)	2 ± 1.6 (19)	41.1 ± 5.8 (20)	32 ± 1.8 (19)	3.7 ± 2.3 (19)
Left Lateral Gastrocnemius	16.8 ± 29.3 (20)	60.1 ± 34 (19)	26 ± 10.6 (20)	17.8 ± 27.7 (20)	1.8 ± 1.6 (19)	39.4 ± 3.9 (20)	32.1 ± 1.1 (18)	2.8 ± 1.7 (18)
Left Medial Gastrocnemius	20 ± 32.5 (20)	64 ± 33.8 (19)	25.8 ± 10.8 (20)	19.7 ± 29.8 (19)	1.9 ± 1.3 (19)	39.7 ± 3.6 (20)	32.4 ± 1 (18)	3.9 ± 1.6 (19)
Left Soleus	12.3 ± 23.3 (20)	55.6 ± 27 (19)	28 ± 8.7 (20)	13.3 ± 22.9 (19)	2.4 ± 1.8 (19)	41.5 ± 4.2 (20)	32 ± 1.4 (19)	2.9 ± 2.3 (17)
Left Tibialis Posterior	11.5 ± 21.8 (20)	58.6 ± 30.2 (19)	27.1 ± 8.5 (20)	14.1 ± 22 (20)	1.4 ± 0.8 (19)	39.5 ± 2.5 (20)	31.9 ± 1.6 (19)	2.3 ± 1.3 (19)

FF: fat fraction; MTR: Magnetisation transfer ratio; FFw: Fat fraction from whole muscle region of interest. Values are mean ± standard deviation (number of values).

Table e3: Baseline individual muscle values in IBM and matched controls

	IBM				Control (IBM)			
Muscle	FF (%)	T2 (ms)	MTR (p.u.)	FFw (%)	FF (%)	T2 (ms)	MTR (p.u.)	FFw (%)
Right Rectus Femoris	19 ± 16.8 (20)	81.3 ± 29.1 (19)	20.6 ± 8.2 (9)	26.8 ± 17.6 (20)	0.6 ± 0.7 (19)	41.8 ± 4.5 (16)	30.7 ± 2.3 (3)	3.1 ± 1.5 (19)
Right Vastus Medialis	31.4 ± 22.7 (20)	101.2 ± 27.6 (19)	16.9 ± 6.6 (9)	37.4 ± 17.5 (20)	1.4 ± 0.7 (19)	43.8 ± 4.3 (17)	31.9 ± 2.4 (5)	2.7 ± 1 (19)
Right Vastus Intermedius	37.6 ± 26.5 (20)	99.4 ± 30.7 (20)	15.5 ± 9.1 (15)	33.9 ± 20.5 (20)	1.1 ± 0.9 (19)	39.3 ± 2.4 (18)	32.8 ± 1.8 (14)	2.7 ± 1.6 (19)
Right Vastus Lateralis	49.2 ± 27.8 (20)	115.9 ± 31.3 (20)	12.8 ± 8.6 (16)	44.1 ± 24 (20)	1.5 ± 0.9 (19)	42.7 ± 3.7 (17)	32 ± 1.2 (18)	3.2 ± 1.1 (19)
Right Semimembranosus	19.6 ± 23.8 (20)	71.2 ± 28.4 (20)	23.6 ± 8.9 (18)	23 ± 22.6 (20)	3.1 ± 2.9 (19)	47.4 ± 6.6 (18)	30.8 ± 1.6 (16)	5.7 ± 2.4 (19)
Right Semitendinosus	19.1 ± 28.3 (20)	68.6 ± 29.1 (20)	26.4 ± 7.5 (17)	23.5 ± 25.8 (20)	1.1 ± 0.6 (19)	43.5 ± 3.5 (18)	32.1 ± 0.8 (17)	4.4 ± 2.5 (19)
Right Biceps Femoris	6.6 ± 10.2 (20)	59.7 ± 17.2 (19)	28.6 ± 4.3 (18)	9.5 ± 8.4 (20)	2.6 ± 1.2 (19)	48 ± 4 (17)	30.5 ± 1.1 (17)	4.1 ± 2.2 (19)
Right Adductor Magnus	34.3 ± 38.5 (20)	89.1 ± 41 (19)	22.3 ± 11 (16)	34.5 ± 32.8 (20)	2.4 ± 3 (19)	44.4 ± 5.7 (18)	31.5 ± 1.7 (17)	4.6 ± 3.3 (19)
Right Sartorius	22.4 ± 24.7 (20)	74.4 ± 24.3 (20)	24.6 ± 5.5 (11)	26.7 ± 22.6 (20)	3.5 ± 3.4 (19)	44.1 ± 5.8 (16)	30.8 ± 1.5 (10)	6.7 ± 2.8 (19)
Right Gracilis	27.4 ± 31.1 (20)	71.8 ± 30.1 (20)	24 ± 8.6 (15)	29.1 ± 26.4 (20)	1.6 ± 1.2 (19)	40.4 ± 3.5 (17)	32.9 ± 1.6 (17)	4.5 ± 2 (17)
Left Rectus Femoris	29.5 ± 25.5 (20)	98.3 ± 39.6 (19)	18.7 ± 7.4 (10)	33.8 ± 22.9 (20)	1 ± 0.7 (19)	39.4 ± 5 (17)	32.6 ± 1.8 (18)	3.3 ± 1.8 (19)
Left Vastus Medialis	37.5 ± 25.5 (20)	114.2 ± 39.1 (19)	16.4 ± 9.3 (13)	39.2 ± 20.7 (20)	1.4 ± 1.1 (19)	42.3 ± 4.2 (19)	32.4 ± 1.2 (18)	3.2 ± 1.2 (19)
Left Vastus Intermedius	38.4 ± 27.5 (20)	119.4 ± 41.4 (18)	17.4 ± 8.1 (13)	38 ± 21.7 (20)	1.4 ± 1 (19)	43.4 ± 5 (19)	31.6 ± 1.7 (18)	2.4 ± 1.1 (19)
Left Vastus Lateralis	46.4 ± 28.5 (20)	108.4 ± 32.1 (19)	14.7 ± 9.2 (14)	44.2 ± 21.1 (20)	2 ± 1 (19)	42 ± 2.1 (19)	31.7 ± 0.9 (18)	3.9 ± 1.3 (19)
Left Semimembranosus	10.9 ± 15.3 (20)	55.8 ± 15.9 (20)	27.6 ± 2.5 (16)	17.5 ± 14.5 (20)	2.8 ± 1.7 (18)	44.5 ± 5.4 (19)	29.9 ± 1.4 (15)	5.1 ± 2.5 (18)
Left Semitendinosus	16.1 ± 26.1 (20)	60.3 ± 24 (20)	28.1 ± 4.5 (15)	21 ± 23.3 (20)	1.4 ± 0.8 (19)	42.3 ± 4.6 (19)	31.4 ± 0.9 (16)	4.8 ± 2 (18)
Left Biceps Femoris	6.1 ± 8.2 (20)	54.9 ± 14.4 (20)	29.6 ± 1.7 (16)	11.4 ± 8.7 (20)	2.9 ± 2.1 (19)	44.8 ± 6.1 (19)	31.4 ± 1.2 (18)	5.3 ± 2.7 (19)
Left Adductor Magnus	33.9 ± 36.4 (20)	87.5 ± 41.5 (19)	20.3 ± 11.4 (14)	29 ± 28.5 (20)	2.5 ± 2.3 (19)	44 ± 4.9 (19)	31.1 ± 1.2 (17)	4.7 ± 3.2 (19)
Left Sartorius	25.8 ± 27.4 (20)	81.5 ± 33.3 (20)	24.7 ± 7 (13)	29.9 ± 25.6 (20)	3.4 ± 3 (19)	43.7 ± 6.2 (19)	31.1 ± 1.4 (15)	7.7 ± 3.3 (19)
Left Gracilis	21.2 ± 23.2 (20)	63.4 ± 22.1 (20)	26.6 ± 5.8 (12)	24.5 ± 19.3 (20)	1.7 ± 1.4 (19)	39.4 ± 3.8 (19)	31.9 ± 1.6 (16)	5 ± 2 (19)
Right Tibialis Anterior	7.1 ± 14.6 (17)	64.2 ± 35 (18)	25.3 ± 7.1 (13)	7.4 ± 10.6 (17)	1 ± 0.5 (18)	38.5 ± 2.5 (19)	29.9 ± 1.2 (15)	1.9 ± 0.9 (17)
Right Peroneus Longus	9 ± 13.4 (17)	65.4 ± 32.5 (18)	26.6 ± 6.6 (18)	12.4 ± 10.6 (17)	1.7 ± 1.5 (18)	41.5 ± 4.8 (18)	31.4 ± 1.6 (15)	4 ± 2.5 (17)
Right Lateral Gastrocnemius	20.9 ± 29 (17)	75.4 ± 37.8 (19)	23.6 ± 9.9 (18)	23.3 ± 25.5 (17)	1.6 ± 1 (18)	43.2 ± 6.6 (19)	31.4 ± 1.2 (16)	3 ± 1.4 (17)
Right Medial Gastrocnemius	53 ± 32 (17)	106.4 ± 29.8 (18)	12.9 ± 9.7 (18)	50.6 ± 25.7 (17)	1.6 ± 0.8 (18)	42.3 ± 4.5 (19)	32 ± 1.7 (16)	3.2 ± 1.4 (17)
Right Soleus	9.2 ± 17.1 (17)	59.8 ± 26.7 (18)	27.5 ± 6.7 (16)	13.1 ± 15.1 (17)	3.1 ± 2.7 (18)	43.9 ± 6.2 (19)	31.5 ± 1.4 (17)	3.9 ± 2.6 (17)
Right Tibialis Posterior	3.8 ± 7.2 (17)	48.6 ± 15.6 (18)	30.8 ± 3.5 (16)	5.8 ± 9.2 (17)	1.3 ± 0.7 (18)	39.8 ± 2.2 (19)	32.6 ± 1.1 (17)	2.1 ± 0.7 (17)
Left Tibialis Anterior	8.9 ± 14.7 (18)	66.6 ± 33.7 (18)	26.2 ± 7 (17)	9.4 ± 11.4 (18)	1.2 ± 0.7 (18)	41.9 ± 5.8 (19)	31.5 ± 1.5 (16)	2.5 ± 1 (17)
Left Peroneus Longus	11 ± 12.3 (18)	64.7 ± 32.2 (19)	25.7 ± 7.7 (19)	15.2 ± 11.4 (18)	2.4 ± 2.1 (18)	42.7 ± 6 (19)	31.2 ± 1.6 (17)	4.6 ± 2.8 (17)
Left Lateral Gastrocnemius	18.5 ± 28.3 (18)	70.7 ± 34.2 (19)	22.9 ± 10.1 (19)	23.2 ± 24.5 (18)	2.1 ± 1.8 (18)	42.2 ± 5.2 (19)	31.5 ± 1.1 (16)	3.5 ± 1.6 (16)
Left Medial Gastrocnemius	54.7 ± 29.7 (18)	111 ± 28.9 (19)	12.5 ± 8.5 (19)	52.9 ± 24.5 (18)	2.9 ± 2.1 (18)	42.1 ± 6.4 (19)	32 ± 0.9 (16)	4.6 ± 1.7 (17)
Left Soleus	14.1 ± 20 (18)	63.6 ± 30.3 (19)	26.4 ± 8.3 (19)	17.8 ± 18.8 (18)	3.2 ± 1.7 (18)	43.2 ± 4.9 (19)	31.6 ± 1.4 (17)	4.1 ± 2.1 (15)
Left Tibialis Posterior	6.5 ± 17.5 (18)	54.3 ± 29.8 (19)	29.1 ± 6.2 (18)	9.3 ± 14.7 (18)	1.6 ± 1 (18)	40.5 ± 2.3 (19)	31.5 ± 1.6 (17)	2.9 ± 1.4 (17)

FF: fat fraction; MTR: Magnetisation transfer ratio; FFw: Fat fraction from whole muscle region of interest. Values are mean ± standard deviation (number of values).

Table e4: Linear regression of quantitative MRI variables at baseline, at thigh and calf level with all groups combined

Thigh			Dependant variable		
			FF	T2	MTR
Independent variable	FF	R		0.92	0.93
		Constant		42.6	31.8
		Slope		1.35	-0.38
	T2	R	0.92		0.95
		Constant	-26		42.2
		Slope	0.63		-0.25
	MTR	R	0.93	0.95	
		Constant	73	155	
		Slope	-2.27	-3.55	

Calf			Dependant variable		
			FF	T2	MTR
Independent variable	FF	R		0.93	0.96
		Constant		41.6	31.7
		Slope		1.19	-0.35
	T2	R	0.93		0.97
		Constant	-29		43.2
		Slope	0.73		-0.28
	MTR	R	0.96	0.97	
		Constant	84	149	
		Slope	-2.61	-3.38	

Linear regression of pair-wise comparison of quantitative MRI parameters on an individual muscle basis at thigh and calf level. For example at thigh level the equation T2 = 42.6 + 0.92 x fat fraction fits data best, with R = 0.92. All quantitative MRI parameters are highly significant ($p<0.001$for all). Strongest correlation is seen between T2 and MTR at both thigh and calf level. Constant and slope are similar at thigh and calf level for equivalent correlations. FF: fat fraction; MTR: magnetisation transfer ratio; R: model fit parameter.

Table e5: Linear regression T2 and MTR in muscles fat fraction in the healthy control range (FF < 4·8% thigh; FF < 4·7% calf) in IBM (A) and CMT1A (B) patients and matched controls

**A**	**Thigh**			**Calf**		
T2	R=0·62, p<0·0001			R=0·52, p<0·0001		
	Co-eff	*Std Error*	p	*Co-eff*	*Std Error*	p
Constant	38·4	0·43	<0·0001	38·0	0·53	<0.0001
IBM	4·0	0·54	<0·0001	3·5	0·57	<0.0001
FF	2·52	0·21	<0·0001	1·78	0·25	<0.0001
MTR	R=0·61, p=<0·0001			R=0·39, p<0·0001		
	Co-eff	*Std Error*	p	*Co-eff*	*Std Error*	p
Constant	32·9	0·16	<0.0001	32·3	0·20	<0·0001
IBM	-1·5	0·19	<0.0001	-1·1	0·21	<0·0001
FF	-0·74	0·07	<0.0001	-0·33	0·09	0·0007

**B**	**Thigh**			**Calf**		
T2	R=0·49, p<0·0001			R=0·59, p<0·0001		
	Co-eff	*Std Error*	*p*	*Co-eff*	*Std Error*	*p*
Constant	38·4	0·29	<0·0001	36·6	0·31	<0·0001
CMT1A	1·0	0·31	0·0008	2·0	0·33	<0·0001
FF	2·07	0·15	<0·0001	1·87	0·17	<0·0001
MTR	R=0·40, p<0·0001			R=0·38, p<0·0001		
	Co-eff	*Std Error*	*p*	*Co-eff*	*Std Error*	*p*
Constant	33·0	0·12	<0·0001	32·8	0·14	<0·0001
CMT1A	-0·3	0·13	0·04	-0·7	0·15	<0·0001
FF	-0·65	0·06	<0.0001	-0·40	0·08	<0·0001

Determinants of T2 and MTR in muscles with normal FF. FF remains strongly correlated to both T2 and MTR, however subject group (patient = 1, control =0) also has a significant effect. R: Overall model correlation coefficient; CMT1A: Charcot-Marie-Tooth disease type 1A; IBM: inclusion body myositis; FF: fat fraction; MTR: magnetisation transfer ratio; Co-eff: partial regression coefficient; p: significance level.

Table e6: Correlation of muscle strength with MRI parameters in each subject group

		IBM			CMT			Control		
		Area	FF	RMA	Area	FF	RMA	Area	FF	RMA
Right Quadriceps	ICC	0.665**	-0.551*	0.809**	0.780**	-0.323	0.791**	0.903**	-0.407*	0.907**
	p	0.001	0.012	0.000	0.000	0.165	0.000	0.000	0.035	0.000
Left Quadriceps	ICC	0.727**	-0.778**	0.917**	0.848**	-0.286	0.860**	0.900**	-0.251	0.905**
	p	0.000	0.000	0.000	0.000	0.222	0.000	0.000	0.207	0.000
Right Hamstrings	ICC	0.702**	-0.561*	0.800**	0.469*	-0.252	0.539*	0.647**	-0.264	0.670**
	p	0.001	0.010	0.000	0.043	0.298	0.017	0.000	0.183	0.000
Left Hamstrings	ICC	0.706**	-0.579**	0.790**	0.587**	-0.295	0.664**	0.635**	-0.247	0.660**
	p	0.001	0.007	0.000	0.008	0.220	0.002	0.000	0.215	0.000
Right Anterior Compartment	ICC	0.780**	-0.509*	0.833**	0.607**	-0.544*	0.775**	0.600**	-0.195	0.622**
	p	0.000	0.037	0.000	0.004	0.013	0.000	0.001	0.339	0.001
Left Anterior Compartment	ICC	0.675**	-0.538*	0.796**	0.606**	-0.634**	0.759**	0.672**	-0.376	0.677**
	p	0.002	0.021	0.000	0.005	0.004	0.000	0.000	0.058	0.000
Right Triceps Surae	ICC	0.476*	-0.626**	0.769**	0.531*	-0.609**	0.719**	0.589**	-0.235	0.613**
	p	0.039	0.007	0.000	0.016	0.004	0.000	0.001	0.248	0.001
Left Triceps Surae	ICC	0.452	-0.556*	0.681**	0.741**	-0.681**	0.834**	0.493**	-0.010	0.540**
	p	0.052	0.017	0.002	0.000	0.001	0.000	0.008	0.963	0.004

IBM: inclusion body myositis; T: Charcot-Marie-Tooth disease; FF: fat fraction; RMA: remaining muscle area (see methods for definition); ICC: intra-class correlation coefficient; * significant correlation p<0.05; ** significant correlation p<0.01.

Table e7: Comparison of outcome measure responsiveness with previous studies in CMT1A

Measure	Baseline	Change	p	SRM
This study, 1 year				
**Mean calf fat fraction (%)**	15·5 ± 24·0	1·22 ± 1·47	0·002	0·83
**CMTES (0-28)**	8·0 ± 5·1	0·3 ± 1·3	ns	0·23
**MRC lower limb**	95·4 ± 15·4	-0·4 ± 3·8	ns	-0·11
**SF-36 PF (0-100%)**	65·3 ± 23·2	-1·9 ± 14·9	ns	-0·12
Verhamme[4], 5 years, 46 patients				
**Adapted CMTNS^ (0-33)**	11·6 ± 4·5	1·5 ± 3·0	0·003	0·49
**Nine-hole peg test (s)**	26·0 ± 18·4	2·0 ± 5·8	0·02	0·35
**Three-point grip (N)**	7 ± 32	-6·3 ± 10·5	<0·001[#]	-0·60
**Ankle dorsiflexion (N)**	190 ± 74	0·1 ± 28·1	ns	0·00
**Ankle plantarflexion (N)**	>250[+]	NA	NA	NA
**Ulnar CMAP (mV)**	4·1 ± 1·5	-0·4 ± -0·7	0·001*	-0·60
Pareyson[6], 2 years, 133 patients (placebo arm)				
**CMTNS (0-36)**	13·9 ± 4·3	0·5 ± 2·7	<0·05	0·19
**CMTES (0-28)**	8·6 ± 3·6	0·5 ± 2·1	<0·05	0·23
**CMT NCS (0-8)**	5·2 ± 1·6	-0·1 ± 1·6	ns	-0·06
**Nine-hole peg test (s)**	23·4 ± 5·7	0·85 ± 2·7	<0·01	0·31
**SF-36 PF (0-100%)**	62·9 ± 25·7	-1·1 ± 15·4	ns	-0·07
**Hand grip (N)**	85·8 ± 38·8	-6·9 ± 20·3	<0·001	-0·34
**Three point pinch (N)**	65·2 ± 29·4	-3·6 ± 18·8	<0·05	-0·19
**Ankle dorsiflexion (N)**	62·8 ± 43·1	-9·8 ± 23·7	<0·001	-0·42
**Ankle plantarflexion (N)**	97·0 ± 59·7	-2·7 ± 47·7	ns	-0·06
**CMAP sum (mV)**	7·1 ± 4·1	0·2 ± 2·9	ns	0·08

Values expressed mean ± standard deviation. Non-significant (versus baseline or control) SRM depicted in grey. #: identical difference seen in controls; *: greater reduction seen in controls (p=0·05); +: measurement limited to 250N; ^: data for this measurement collected retrospectively; NA: not available; ns: not significant; SRM: standardised response mean, calculated from published data as mean change/standard deviation change. Standard deviation in Pareyson study calculated from published 95% confidence interval by standard statistical formulae: 95%CI = mean - 1.96 s.d. to mean + 1.96 s.d.; standard error mean = s.d./√n

Figure e1: Correlation of fat fraction with disease duration in IBM and CMT1A patients

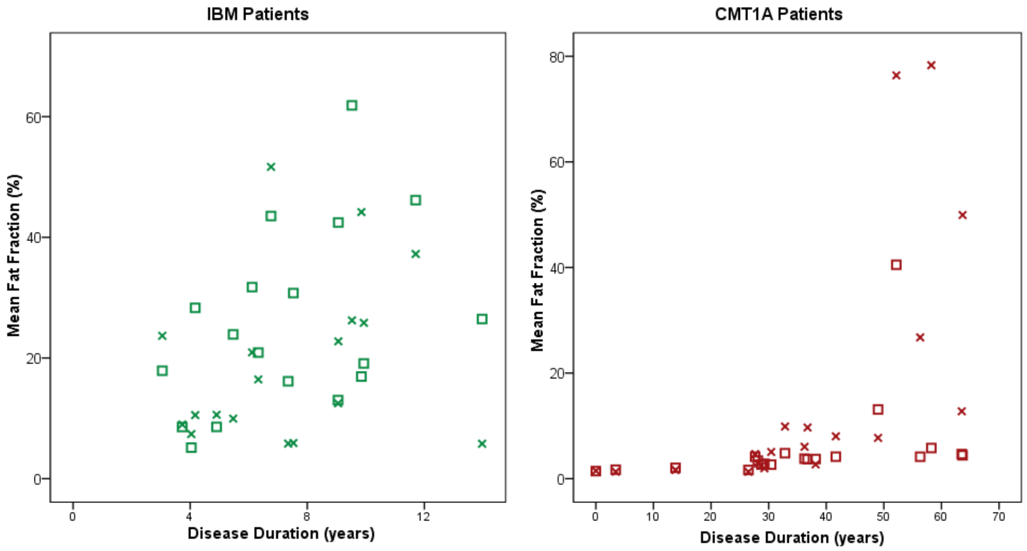

Scatter plot of disease duration versus thigh-level (□) and calf-level (x) fat fraction in IBM and CMT1A patients. In IBM patients positive correlation between disease duration and mean fat fraction is seen at thigh level (rho=0.50,p=0.03) but not calf level (rho=0.27, p=0.28). In CMT1A patients, strong positive correlations at both levels are seen (thigh: rho=0.81,p<0.0001 ; calf: rho=0.89, p<0.0001). The relationship between disease duration and fat fraction appears non-linear in the CMT1A group.

Figure e2: Correlation of MRI measured remaining muscle area with isometric muscle strength

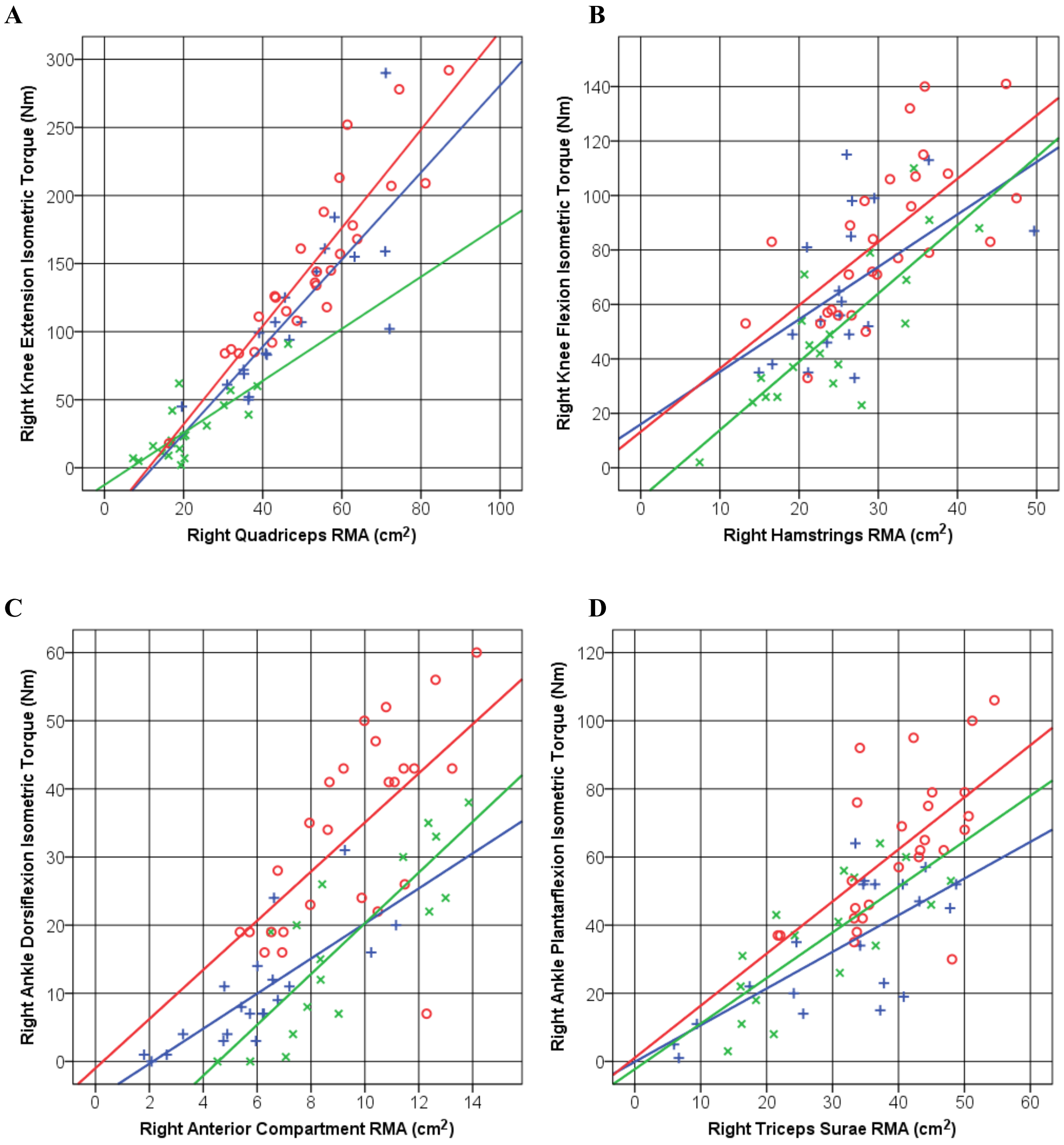

MRI measured RMA of lower limb muscle groups show strong correlation with corresponding strength in IBM (x), CMT1A (+) and controls (o).

A: Right quadriceps. IBM: rho=0.81, p<0.0001; CMT: rho=0.79, p<0.0001; controls: rho=0.91, p<0.0001

B: Right hamstrings. IBM: rho=0.80, p<0.0001; CMT: rho=0.54, p=0.02; controls: rho=0.67, p<0001

C: Right anterior compartment. IBM: rho=0.83, p<0.0001; CMT: rho=0.78, p<0.0001; controls: rho=0.62, p=0.0007

D: Right triceps surae. IBM: rho=0.77, p<0.0001; CMT: rho=0.72, p<0.0001; controls: rho=0.61, p=0.0009

Figure e3: Correlation of calf fat fraction with severity score (CMTES) in CMT1A patients

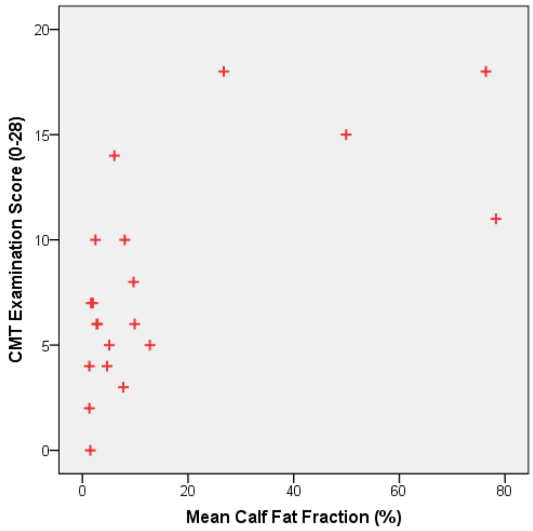

There is significant correlation between calf muscle fat fraction and CMTES in CMT1A patients (rho=0.63, p=0.003). The relationship appears non-linear.

Supplementary references

1 Hilton-Jones D, Miller A, Parton M, Holton J, Sewry C, Hanna MG. Inclusion body myositis: MRC Centre for Neuromuscular Diseases, IBM workshop, London, 13 June 2008. Neuromuscul Disord 2010; **20**: 142–7.
2 Reilly MM, Murphy SM, Laurá M. Charcot-Marie-Tooth disease. J Peripher Nerv Syst JPNS 2011; **16**: 1–14.
3 Pareyson D, Reilly MM, Schenone A, et al. Ascorbic acid in Charcot-Marie-Tooth disease type 1A (CMT-TRIAAL and CMT-TRAUK): a double-blind randomised trial. Lancet Neurol 2011; **10**: 320–8.
4 Morrow JM, Sinclair CDJ, Fischmann A, et al. Reproducibility, and age, body-weight and gender dependency of candidate skeletal muscle MRI outcome measures in healthy volunteers. Eur Radiol 2014; **24**: 1610–20.
5 O'Brien MD. Aids to the Examination of the Peripheral Nervous System. Elsevier Health Sciences, 2000.
6 Glover GH, Schneider E. Three-point Dixon technique for true water/fat decomposition with B0 inhomogeneity correction. Magn Reson Med Off J Soc Magn Reson Med Soc Magn Reson Med 1991; **18**: 371–83.
7 Smith SM, Jenkinson M, Woolrich MW, et al. Advances in functional and structural MR image analysis and implementation as FSL. NeuroImage 2004; **23 Suppl 1**: S208–19.
8 Stollberger R, Wach P. Imaging of the active B1 field in vivo. Magn Reson Med Off J Soc Magn Reson Med Soc Magn Reson Med 1996; **35**: 246–51.
9 Barker GJ, Tofts PS, Gass A. An interleaved sequence for accurate and reproducible clinical measurement of magnetization transfer ratio. Magn Reson Imaging 1996; **14**: 403–11.
10 Sinclair CDJ, Morrow JM, Hanna MG, et al. Correcting radiofrequency inhomogeneity effects in skeletal muscle magnetisation transfer maps. NMR Biomed 2012; **25**: 262–70.
